# Protein Intake and Mortality in Older Adults With Chronic Kidney Disease

**DOI:** 10.1001/jamanetworkopen.2024.26577

**Published:** 2024-08-07

**Authors:** Adrián Carballo-Casla, Carla Maria Avesani, Giorgi Beridze, Rosario Ortolá, Esther García-Esquinas, Esther Lopez-Garcia, Lu Dai, Michelle M. Dunk, Peter Stenvinkel, Bengt Lindholm, Juan Jesús Carrero, Fernando Rodríguez-Artalejo, Davide Liborio Vetrano, Amaia Calderón-Larrañaga

**Affiliations:** 1 Aging Research Center, Department of Neurobiology, Care Sciences and Society, Karolinska Institutet and Stockholm University, Stockholm, Sweden; 2 Center for Networked Biomedical Research in Epidemiology and Public Health, Madrid, Spain; 3 Department of Clinical Science Intervention and Technology, Division of Renal Medicine, Baxter Novum, Karolinska Institutet, Stockholm, Sweden; 4 Department of Preventive Medicine and Public Health, Universidad Autónoma de Madrid, Madrid, Spain; 5 National Center of Epidemiology, Instituto de Salud Carlos III, Madrid, Spain; 6 IMDEA Food Institute, Campus of International Excellence, Madrid, Spain; 7 Department of Medical Epidemiology and Biostatistics, Karolinska Institutet, Stockholm, Sweden; 8 Division of Nephrology, Department of Clinical Sciences, Karolinska Institutet, Danderyd Hospital, Stockholm, Sweden; 9 Stockholm Gerontology Research Center, Stockholm, Sweden

## Abstract

**Importance:**

Avoiding high protein intake in older adults with chronic kidney disease (CKD) may reduce the risk of kidney function decline, but whether it can be suboptimal for survival is not well known.

**Objective:**

To estimate the associations of total, animal, and plant protein intake with all-cause mortality in older adults with mild or moderate CKD and compare the results to those of older persons without CKD.

**Design, Setting, and Participants:**

Data from 3 cohorts (Study on Cardiovascular Health, Nutrition and Frailty in Older Adults in Spain 1 and 2 and the Swedish National Study on Aging and Care in Kungsholmen [in Sweden]) composed of community-dwelling adults 60 years or older were used. Participants were recruited between March 2001 and June 2017 and followed up for mortality from December 2021 to January 2024. Those with no information on diet or mortality, with CKD stages 4 or 5, or undergoing kidney replacement therapy and kidney transplant recipients were excluded. Data were originally analyzed from June 2023 to February 2024 and reanalyzed in May 2024.

**Exposures:**

Cumulative protein intake, estimated via validated dietary histories and food frequency questionnaires.

**Main Outcomes and Measures:**

The study outcome was 10-year all-cause mortality, ascertained with national death registers. Chronic kidney disease was ascertained according to estimated glomerular filtration rates, urine albumin excretion, and diagnoses from medical records.

**Results:**

The study sample consisted of 8543 participants and 14 399 observations. Of the 4789 observations with CKD stages 1 to 3, 2726 (56.9%) corresponded to female sex, and mean (SD) age was 78.0 (7.2) years. During the follow-up period, 1468 deaths were recorded. Higher total protein intake was associated with lower mortality among participants with CKD; adjusted hazard ratio (HR) for 1.00 vs 0.80 g/kg/d was 0.88 (95% CI, 0.79-0.98); for 1.20 vs 0.80 g/kg/d, 0.79 (95% CI, 0.66-0.95); and for 1.40 vs 0.80 g/kg/d, 0.73 (95% CI, 0.57-0.92). Associations with mortality were comparable for plant and animal protein (HRs, 0.80 [95% CI, 0.65-0.98] and 0.88 [95% CI, 0.81-0.95] per 0.20-g/kg/d increment, respectively) and for total protein intake in participants younger than 75 years vs 75 years or older (HRs, 0.94 [95% CI, 0.85-1.04] and 0.91 [95% CI, 0.85-0.98] per 0.20-g/kg/d increment in total protein intake, respectively). However, the hazards were lower among participants without CKD than in those with CKD (HRs, 0.85 [95% CI, 0.79-0.92] and 0.92 [95% CI, 0.86-0.98] per 0.20-g/kg/d increment, respectively; *P* = .02 for interaction).

**Conclusions and Relevance:**

In this multicohort study of older adults, higher intake of total, animal, and plant protein was associated with lower mortality in participants with CKD. Associations were stronger in those without CKD, suggesting that the benefits of proteins may outweigh the downsides in older adults with mild or moderate CKD.

## Key Points

**Question:**

What are the associations of total, animal, and plant protein intake with all-cause mortality in older adults with mild or moderate chronic kidney disease (CKD)?

**Findings:**

In this cohort study of 8543 community-dwelling adults 60 years and older, higher intake of total, animal, and plant protein was associated with lower mortality in participants with mild or moderate CKD. Associations were larger among participants without CKD.

**Meaning:**

These findings suggest that the benefits of proteins may outweigh the downsides in older adults with mild or moderate CKD, in whom disease progression may play a more limited role in survival.

## Teaser

This multicohort study estimates the associations of total, animal, and plant protein intake with all-cause mortality in older adults with mild or moderate chronic kidney disease (CKD) and compares the results with those of older adults without kidney disease.

## Introduction

Aging is characterized by multiple behavioral and physiological changes across organs and systems that impair protein utilization and increase its requirements.^1,2^ On the one hand, protein synthesis is reduced because of a shortage of available nutrients due to loss of appetite, sedentary behavior, and insulin and protein anabolic resistance.^1,2^ On the other hand, protein degradation, increased oxidative modification of proteins, and accumulation of inflammatory diseases lead to an increased need for protein.^1,2^

To preserve physical function and support good health and recovery from illness, protein recommendations for healthy older individuals lie between 1.00 and 1.20 g/kg of actual body weight per day (g/kg/d).^1,2,3^ Additional increases may be warranted for those with acute and multiple chronic diseases and those with severe illness, injuries, or malnutrition.^1,2^

While older adults may need more protein than younger persons, higher protein intake could accelerate disease progression among those with chronic kidney disease (CKD), a prevalent condition in older adults that often has no cure and high morbidity and mortality.^4,5^ According to current guidelines, adults with mild CKD (stages 1 and 2) are advised to avoid high protein intake (>1.30 g/kg/d), and those with moderate or severe CKD (stages 3-5 not receiving dialysis) are advised to restrict protein intake to 0.60 to 0.80 g/kg/d.^4,5,6^ Such a regimen of lower protein intake has been shown to slow CKD progression rates and improve metabolic derangements in persons with CKD stages 4 and 5 not receiving dialysis.^7,8,9^

Despite the benefits in adults of all ages with severe CKD, insufficient evidence of the overall health impact of limiting protein intake in older persons with mild or moderate CKD, and whether this impact is different in older adults without CKD, is available.^4,5,6^ The latest guidelines leave the door open for higher protein intake targets in those with underlying conditions, such as frailty and sarcopenia, but more data are needed to make specific recommendations.^5^ Observational studies on mortality in older persons with CKD are often small to moderate in size, are conducted in a single setting, and use point-in-time estimates of protein intake,^10,11,12^ while randomized clinical trials commonly include participants with CKD of all ages and lack power to analyze protein intake modification in those who are older.^13,14^

The role of protein sources in older adults with CKD is also of interest. Plant protein might have a lower impact on remaining nephrons, mitigate glomerular hyperfiltration, reduce proteinuria, preserve kidney function, and protect from metabolic derangements, while animal-based protein has higher biological value and anabolic potential, so its intake may translate into improved nutritional status.^4,5,6,15,16^ Nevertheless, whether a diet that is high in plant protein could provide the benefits of higher protein intake without the known downsides in older adults with CKD remains to be investigated.

In this study, we pooled longitudinal data from 3 cohorts to estimate the associations of total, animal, and plant protein intake with all-cause mortality in older persons with mild or moderate CKD and compared the results with those of older adults without CKD. To examine age-related physiological changes and allow for better risk stratification, we also analyzed the differences between participants younger than 75 years vs 75 years or older.

## Methods

### Study Design and Participants

This multicohort study follows the Strengthening the Reporting of Observational Studies in Epidemiology (STROBE) reporting guideline for observational cohort studies. Three studies were included in the analyses. The Study on Cardiovascular Health, Nutrition and Frailty in Older Adults in Spain (Seniors-ENRICA) 1 and 2 are cohorts of randomly sampled, community-dwelling individuals in Spain 60 years and older and 65 years and older, respectively.^17,18^ To maximize the use of dietary variables and others, we took data from 4 waves of Seniors-ENRICA 1 (March 2008 to September 2010, February to November 2012, November 2014 to June 2015, and January to July 2017) and 3 waves of Seniors-ENRICA 2 (December 2015 to June 2017, September 2018 to October 2019, and November 2021 to February 2023). The Clinical Research Ethics Committee of the La Paz University Hospital in Madrid approved the research protocols, and all participants gave written informed consent at each study visit.

The Swedish National Study on Aging and Care in Kungsholmen (SNAC-K) is a longitudinal, community-based cohort of randomly sampled adults 60 years or older living in Stockholm, Sweden.^19,20^ All participants attended examinations in March 2001 to August 2004 and February 2007 to October 2010, and those 78 years and older were also assessed in November 2004 to May 2007. SNAC-K was approved by the Regional Ethical Review Board in Stockholm, and written informed consent was obtained from participants or their next of kin.

### Study Variables

#### Diet

Habitual food consumption in the previous year was obtained with an interviewer-administered, validated electronic dietary history in 3 of the 4 waves of Seniors-ENRICA 1 and in 2 of the 3 waves of Seniors-ENRICA 2.^21^ To convert food consumption into nutrients, the dietary history used data from Spanish and other standard food composition tables.^21^ In all SNAC-K waves, dietary data were collected with a self-administered, semiquantitative, validated food frequency questionnaire that consisted of 98 foods and beverages.^22^ Household measures and food composition tables from the Swedish National Food Agency were used to estimate nutrient intake.^22^

Proteins were deemed to have plant or animal origin according to the foods from which they came. Cereal, legume, nut, and other vegetable proteins were considered plant proteins, while dairy, meat, egg, fish, and other animal proteins were considered of animal origin.

#### Chronic Kidney Disease

In all cohorts, the estimated glomerular filtration rate (eGFR) was calculated using the Berlin Initiative Study equation, which is specifically tailored for older adults: 3736 × Serum Creatinine Level ^−0.87^ × Age ^−0.95^ × 0.82 (if female).^23^ For participants from the Seniors-ENRICA cohorts who provided spot urine samples, albumin level of at least 20 mg/L (to convert to g/L, divide by 1000) was used as a measure of kidney damage, as it has shown high specificity for urinary albumin excretion of at least 30 mg in 24 hours.^24^ In addition, we used inpatient and outpatient medical records in SNAC-K, as well as primary care records in Seniors-ENRICA 2. We also used information on deaths with CKD between data collection waves in Seniors-ENRICA 1 and SNAC-K, and we identified those participants undergoing kidney replacement therapy and kidney transplant via *International Statistical Classification of Diseases, Tenth Revision,* codes. We defined CKD as an eGFR of less than 60 mL/min/1.73 m^2^, high urine albumin level, an antemortem or postmortem medical diagnosis, kidney replacement therapy, or kidney transplant. Participants with CKD were grouped into stages 1 to 5 from the Kidney Disease: Improving Global Outcomes guidelines.^5^

#### Mortality

In the Seniors-ENRICA cohorts, mortality was ascertained with the Spanish National Death Index. In the SNAC-K cohort, such information was available from the Swedish Cause of Death Register. Data were available until December 2021 in SNAC-K and until January 2024 in Seniors-ENRICA 1 and 2.^17,23^

#### Other Variables

Potential confounders included sex, age, living arrangement, previous occupation, educational level, tobacco smoking, light and moderate-to-vigorous physical activity, body mass index, diabetes, cardiovascular disease, chronic lung disease, musculoskeletal disease, cancer, depression and mood disorders, and intake of energy, monounsaturated fat, sugar, alcohol, and sodium. Race and ethnicity were not considered due to data not being available in Seniors-ENRICA 2 and SNAC-K. Information on potential confounders and their data sources can be found in eMethods 1 in Supplement 1.

### Analytical Sample

Of 10 149 recruited participants, we excluded 1566 who had no information on diet and 1 who had no information on mortality. We additionally excluded participants with CKD stages 4 or 5 (n = 30), those undergoing kidney replacement therapy (n = 7), and kidney transplant recipients (n = 2).^4,6^ Hence, the analytical sample comprised 8543 persons and 14 399 observations (4789 with CKD and 9610 without). The same criteria were used to exclude participant observations during the follow-up (eFigure 1 in Supplement 1).

### Statistical Analysis

#### Main Analyses

Data were originally analyzed from June 2023 to February 2024 and reanalyzed in May 2024. Associations of protein intake with mortality were summarized with hazard ratios (HRs) and 95% CIs and estimated with Cox proportional hazards regression. To represent longer-term dietary intake and minimize within-person variation, we implemented the Andersen and Gill^25^ model and set up the data so that there was 1 observation per time interval for each participant. For each observation, we used the cumulative mean of protein intake and continuous potential confounders and the most recent information on CKD and categorical potential confounders.^26^ All models were adjusted for cohort and the previously mentioned sociodemographic, lifestyle, morbidity, and dietary variables. To increase comparability of results across cohorts, participants were censored at 10 years of follow-up.

Protein intake was expressed as grams per kilogram of body weight per day.^1,2,5,6^ To better capture nonlinear trends and minimize power loss, total protein intake was rounded to the nearest 0.05 and modeled as a restricted cubic spline.^27^ Hazard ratios were evaluated at equally spaced intervals between 0.8 and 1.6 g/kg/d of total protein intake in the Tables and at all the distinct observed values in the Figures. We considered 0.8 g/kg/d the protein intake reference value in both Tables and Figures.^1,2,5,6^ We also modeled total protein intake as a linear variable (per 0.2-g/kg/d increment). Animal and plant protein intake were operationalized using similar procedures, as were the main animal and plant protein sources (ie, dairy, meat, fish, and cereal).

To account for incomplete information in the datasets, we used multiple imputation by chained equations (eMethods 2 in Supplement 1). The number of participant observations with missing data for each variable, collection wave, and cohort can be found in eTable 1 in Supplement 1.

#### Interactions and Ancillary Analyses

Hazard ratios and 95% CIs were obtained from models with 2-way multiplicative interactions between total protein intake and CKD and 3-way multiplicative interactions among protein intake, CKD, and age (<75 vs ≥75 years). Differences in the strength of study associations across subgroups were evaluated with *P* values for interaction, obtained from Wald tests of linear hypotheses. Additional interactions among total protein intake, CKD, and cohort and among the former 2 variables and sex were explored in ancillary analyses. Beyond total protein intake, we built models integrating multiplicative interactions between animal protein intake and CKD and between plant protein intake and CKD (and age, as appropriate). All models included both main effects and interaction terms, significance level was set at α<.05, and hypothesis tests were 2 sided. We also assessed how changes in protein intake (from baseline to the nearest available follow-up) and the proportion of plant protein were associated with mortality. To test the robustness of our results, we conducted 15 sensitivity analyses, which are described in detail in eMethods 3 in Supplement 1. All statistical analyses were performed using Stata, version 18.0 (StataCorp LLC).

## Results

### Descriptive and Outcome Data

Characteristics of 14 399 participant observations stratified by CKD are shown in Table 1, of which 4789 had CKD. A total of 2726 observations with CKD (56.9%) corresponded with female participants and 2063 (43.1%) with male participants. Mean (SD) age was 78.0 (7.2) years, and CKD severity distribution was as follows: 49 participants with CKD (1.0%) were in stage 1, 726 (15.2%) in stage 2, 3323 (69.4%) in stage 3A, and 691 (14.4%) in stage 3B. Total mean (SD) protein intake for participant observations with CKD was 1.15 (0.37) g/kg/d. Participant observations with the frailty phenotype^32^ constituted 452 (9.4%) of those with CKD and 306 (3.2%) of those without CKD (data not shown). Characteristics of participant observations stratified by CKD and age and by CKD and cohort are shown in eTables 2 and 3 in Supplement 1, respectively.

**Table 1.** Characteristics of the Participant Observations, Stratified by CKD

Characteristic	Participant group^a^	
	With CKD (n = 4789)	Without CKD (n = 9610)
Sociodemographic		
Sex		
Male	2063 (43.1)	4352 (45.3)
Female	2726 (56.9)	5258 (54.7)
Age, mean (SD), y	78.0 (7.20)	70.0 (5.8)
Living alone	2919 (61.0)	7132 (74.2)
Previous occupation: manual worker	1913 (39.9)	3934 (40.9)
Educational level		
Primary or less	1998 (41.7)	4268 (44.4)
Secondary	1649 (34.4)	2737 (28.5)
University	1143 (23.9)	2605 (27.1)
Lifestyle		
Tobacco smoking		
Never	2574 (53.7)	4860 (50.6)
Former	1810 (37.8)	3660 (38.1)
Current	406 (8.5)	1090 (11.3)
Light physical activity		
Never	451 (9.4)	385 (4.0)
Less than monthly	419 (8.7)	471 (4.9)
Monthly	938 (19.6)	1887 (19.6)
Weekly	1789 (37.4)	4490 (46.7)
Daily	1193 (24.9)	2377 (24.7)
Moderate-to-vigorous physical activity		
Never	2532 (52.9)	3303 (34.4)
Less than monthly	795 (16.6)	1645 (17.1)
Monthly	673 (14.1)	2086 (21.7)
Weekly	599 (12.5)	2077 (21.6)
Daily	190 (4.0)	500 (5.2)
Body mass index, mean (SD)	27.4 (4.6)	27.5 (4.2)
Morbidity variables		
Chronic kidney disease stage		
1	49 (1.0)	NA
2	726 (15.2)	NA
3A	3323 (69.4)	NA
3B	691 (14.4)	NA
Diabetes	997 (20.8)	1539 (16.0)
Cardiovascular disease	1157 (24.2)	1020 (10.6)
Chronic lung disease	606 (12.7)	1159 (12.1)
Musculoskeletal disease	2276 (47.5)	4721 (49.1)
Cancer	647 (13.5)	828 (8.6)
Depression and mood disorders	520 (10.9)	1037 (10.8)
Dietary intake, mean (SD)		
Energy, g/kg/d	27.96 (8.41)	27.42 (7.26)
Total protein, g/kg/d	1.15 (0.37)	1.18 (0.34)
Animal protein, g/kg/d	0.78 (0.29)	0.79 (0.26)
Plant protein, g/kg/d	0.37 (0.14)	0.39 (0.14)
Monounsaturated fat, g/kg/d	0.47 (0.17)	0.48 (0.17)
Sugar, g/kg/d	1.31 (0.54)	1.26 (0.49)
Alcohol, g/kg/d	0.12 (0.16)	0.13 (0.17)
Sodium, mg/kg/d	37.56 (14.52)	36.67 (13.21)

Abbreviations: CKD, chronic kidney disease; NA, not applicable.

^a^ Unless otherwise indicated, data are expressed as No. (%) of observations. Numbers are rounded averages across the 10 multiply imputed datasets and may not total 4789 for observations with CKD or 9610 for observations without CKD. Percentages have been rounded and may not total 100.

### Main Results

**Figure 1.**
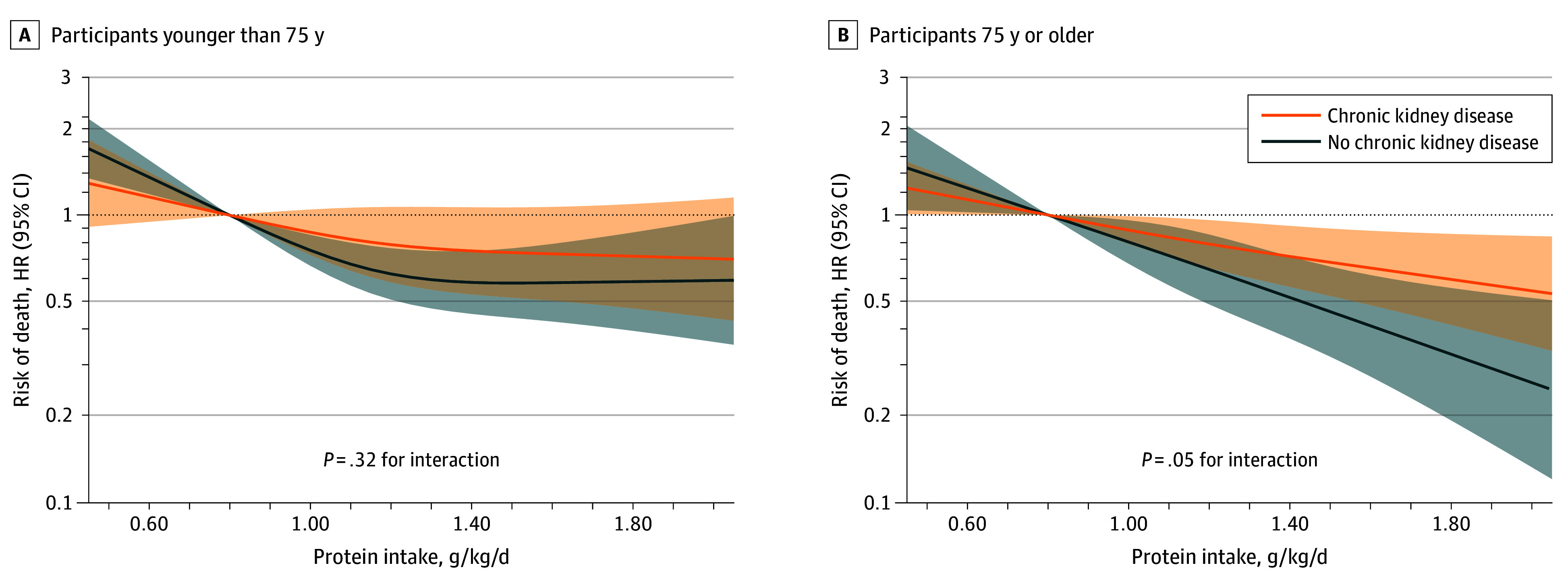
Association of Total Protein Intake With 10-Year All-Cause Mortality, Stratified by Chronic Kidney Disease and Age Analyses used Cox proportional hazards regression models. Protein intake was modeled as a 3-knot restricted cubic spline. Hazard ratios (HRs) and 95% CIs were plotted for protein intake above the 1st percentile and below the 99th percentile and obtained from models with interaction terms among protein intake, chronic kidney disease, and age. Models were adjusted for cohort, sex, age, living arrangement, previous occupation, educational level, tobacco smoking, light physical activity, moderate-to-vigorous physical activity, body mass index, diabetes, cardiovascular disease, chronic lung disease, musculoskeletal disease, cancer, depression and mood disorders, and intake of energy, monounsaturated fat, sugar, alcohol, and sodium.

After a maximum follow-up of 10.0 years, 1468 participants died. As shown in Table 2, higher total protein intake was associated with lower mortality among the participants with CKD. For 1.00 vs 0.80 g/kg/d total protein intake, the HR was 0.88 (95% CI, 0.79-0.98); for 1.20 vs 0.80 g/kg/d, 0.79 (95% CI, 0.66-0.95); for 1.40 vs 0.80 g/kg/d, 0.73 (95% CI, 0.57-0.92); and for 1.60 vs 0.80 g/kg/d, 0.67 (95% CI, 0.51-0.89). Associations were consistent in the participants younger than 75 years (HR, 0.94 [95% CI, 0.85-1.04]) vs 75 years or older (HR, 0.91 [95% CI, 0.85-0.98]) per 0.20-g/kg/d increment; *P* for interaction = .51) (Table 3), with no evidence of departure from linearity (Figure 1).

**Table 2.** Associations of Total, Animal, and Plant Protein Intake With 10-Year All-Cause Mortality, Stratified by CKD

Protein intake	Participant group, HR (95% CI)^a^	
	With CKD	Without CKD
No. of deaths/observations	838/4789	630/9610
Total, g/kg/d		
0.80	1 [Reference]	1 [Reference]
1.00	0.88 (0.79-0.98)	0.77 (0.69-0.87)
1.20	0.79 (0.66-0.95)	0.63 (0.53-0.77)
1.40	0.73 (0.57-0.92)	0.56 (0.44-0.71)
1.60	0.67 (0.51-0.89)	0.51 (0.38-0.69)
Per 0.20 increment	0.92 (0.86-0.98)	0.85 (0.79-0.92)
Animal, g/kg/d		
0.60	1 [Reference]	1 [Reference]
0.75	0.87 (0.80-0.95)	0.88 (0.81-0.97)
0.90	0.79 (0.69-0.90)	0.82 (0.71-0.94)
1.05	0.73 (0.61-0.86)	0.78 (0.65-0.93)
1.20	0.68 (0.54-0.84)	0.74 (0.58-0.95)
Per 0.20 increment	0.88 (0.81-0.95)	0.89 (0.82-0.98)
Plant, g/kg/d		
0.25	1 [Reference]	1 [Reference]
0.35	0.89 (0.76-1.03)	0.70 (0.59-0.82)
0.45	0.77 (0.61-0.98)	0.54 (0.42-0.70)
0.55	0.67 (0.49-0.92)	0.46 (0.34-0.63)
0.65	0.58 (0.38-0.88)	0.39 (0.26-0.59)
Per 0.20 increment	0.80 (0.65-0.98)	0.61 (0.50-0.76)

Abbreviations: CKD, chronic kidney disease; HR, hazard ratio.

^a^ Calculated with Cox proportional hazards regression models. Protein intake was modeled as a continuous variable (per 0.20 g/kg/d) or a 3-knot restricted cubic spline otherwise. Hazard ratios (95% CIs) were obtained from models with multiplicative interaction terms between protein intake and CKD. Models on protein sources integrated multiplicative interactions between animal protein intake and CKD and between plant protein intake and CKD. Models were adjusted for cohort, sex, age, living arrangement, previous occupation, educational level, tobacco smoking, light physical activity, moderate-to-vigorous physical activity, body mass index, diabetes, cardiovascular disease, chronic lung disease, musculoskeletal disease, cancer, depression and mood disorders, and intake of energy, monounsaturated fat, sugar, alcohol, and sodium.

**Table 3.** Associations of Total, Animal, and Plant Protein Intake With 10-Year All-Cause Mortality, Stratified by CKD and Age

Protein intake	Participant group, HR (95% CI)^a^			
	With CKD		Without CKD	
	Aged <75 y	Aged ≥75 y	Aged <75 y	Aged ≥75 y
No. of deaths/observations	157/1622	681/3167	398/7734	232/1876
Total, g/kg/d				
0.80	1 [Reference]	1 [Reference]	1 [Reference]	1 [Reference]
1.00	0.87 (0.73-1.05)	0.89 (0.79-0.99)	0.75 (0.67-0.85)	0.80 (0.68-0.96)
1.20	0.79 (0.58-1.07)	0.79 (0.65-0.96)	0.62 (0.51-0.77)	0.65 (0.49-0.85)
1.40	0.75 (0.53-1.07)	0.72 (0.56-0.92)	0.58 (0.45-0.75)	0.52 (0.37-0.72)
1.60	0.73 (0.50-1.07)	0.65 (0.48-0.88)	0.58 (0.43-0.79)	0.41 (0.27-0.62)
Per 0.20 increment	0.94 (0.85-1.04)	0.91 (0.85-0.98)	0.88 (0.81-0.96)	0.81 (0.74-0.90)
Animal, g/kg/d				
0.60	1 [Reference]	1 [Reference]	1 [Reference]	1 [Reference]
0.75	1.00 (0.83-1.20)	0.84 (0.77-0.93)	0.88 (0.79-0.97)	0.91 (0.78-1.06)
0.90	0.94 (0.73-1.22)	0.75 (0.65-0.86)	0.83 (0.71-0.98)	0.77 (0.62-0.97)
1.05	0.86 (0.63-1.18)	0.69 (0.57-0.83)	0.84 (0.69-1.03)	0.63 (0.46-0.86)
1.20	0.78 (0.52-1.17)	0.64 (0.50-0.82)	0.86 (0.66-1.11)	0.51 (0.32-0.81)
Per 0.20 increment	0.93 (0.82-1.06)	0.87 (0.80-0.95)	0.93 (0.84-1.03)	0.83 (0.73-0.95)
Plant, g/kg/d				
0.25	1 [Reference]	1 [Reference]	1 [Reference]	1 [Reference]
0.35	0.69 (0.54-0.88)	0.94 (0.80-1.09)	0.69 (0.57-0.83)	0.67 (0.53-0.86)
0.45	0.59 (0.42-0.84)	0.82 (0.64-1.05)	0.54 (0.41-0.71)	0.52 (0.37-0.73)
0.55	0.60 (0.39-0.92)	0.68 (0.48-0.94)	0.46 (0.33-0.65)	0.45 (0.30-0.67)
0.65	0.63 (0.36-1.10)	0.55 (0.36-0.86)	0.40 (0.26-0.63)	0.39 (0.23-0.67)
Per 0.20 increment	0.78 (0.57-1.07)	0.81 (0.65-1.00)	0.61 (0.48-0.77)	0.62 (0.47-0.83)

Abbreviations: CKD, chronic kidney disease; HR, hazard ratio.

^a^ Calculated with Cox proportional hazards regression models. Protein intake was modeled as a continuous variable (per 0.20 g/kg/d) or a 3-knot restricted cubic spline otherwise. Hazard ratios (95% CIs) were obtained from models with multiplicative interaction terms between protein intake and CKD. Models on protein sources integrated multiplicative interactions between animal protein intake and CKD and between plant protein intake and CKD. Models were adjusted for cohort, sex, age, living arrangement, previous occupation, educational level, tobacco smoking, light physical activity, moderate-to-vigorous physical activity, body mass index, diabetes, cardiovascular disease, chronic lung disease, musculoskeletal disease, cancer, depression and mood disorders, and intake of energy, monounsaturated fat, sugar, alcohol, and sodium.

**Figure 2.**
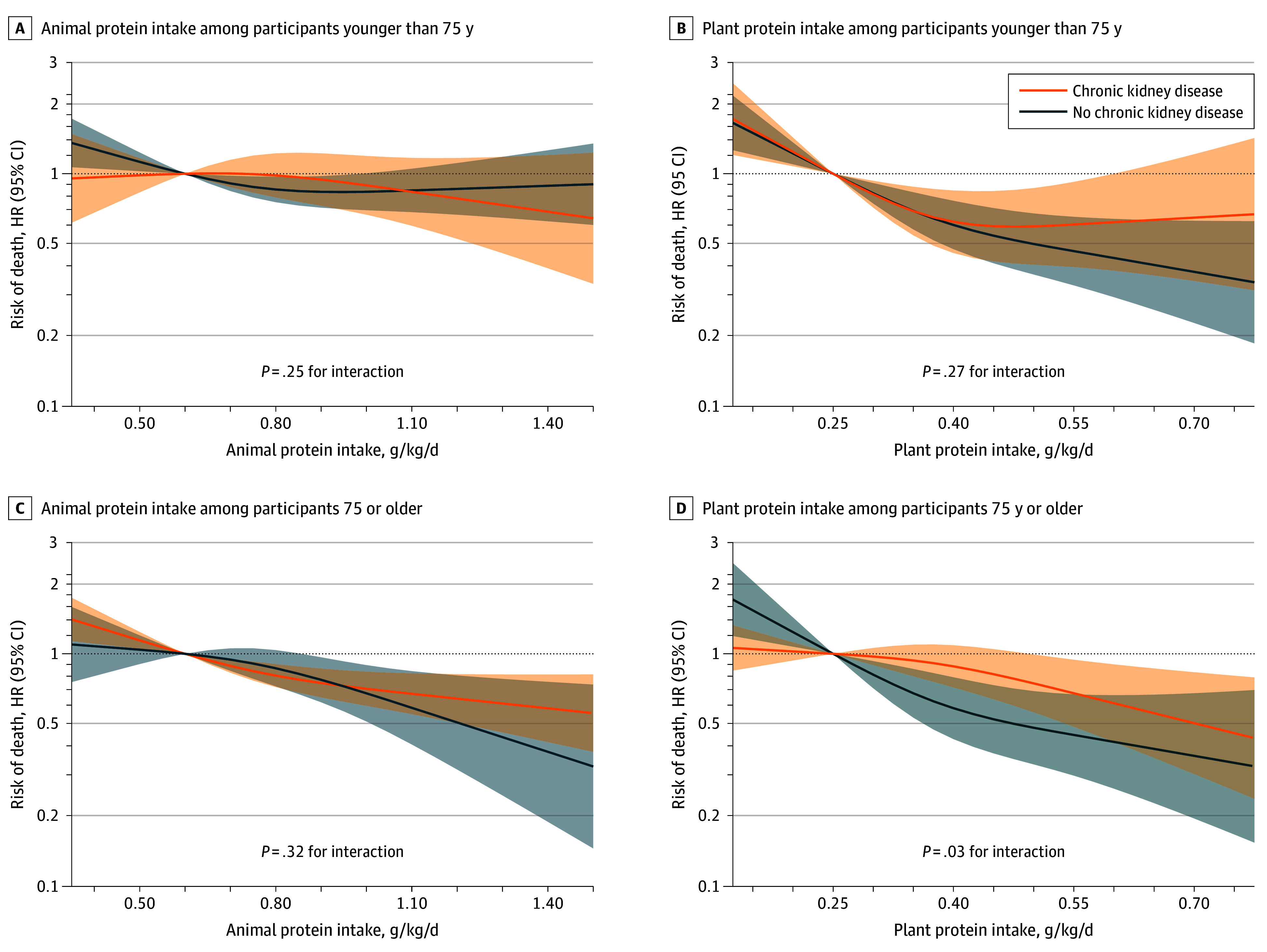
Associations of Animal and Plant Protein Intake With 10-Year All-Cause Mortality, Stratified by Chronic Kidney Disease and Age Analyses used Cox proportional hazards regression models. Protein intake was modeled as a 3-knot restricted cubic spline. Hazard ratios (HRs) and 95% CIs were plotted for protein intake above the 1st percentile and below the 99th percentile and obtained from models integrating interaction terms among animal protein intake, chronic kidney disease, and age and among plant protein intake, chronic kidney disease, and age. Models were adjusted for cohort, sex, age, living arrangement, previous occupation, educational level, tobacco smoking, light physical activity, moderate-to-vigorous physical activity, body mass index, diabetes, cardiovascular disease, chronic lung disease, musculoskeletal disease, cancer, depression and mood disorders, and intake of energy, monounsaturated fat, sugar, alcohol, and sodium.

When examining protein sources among participants with CKD, plant protein (HR, 0.80 [95% CI, 0.65-0.98]) showed a comparable association with mortality to animal protein (HR, 0.88 [95% CI, 0.81-0.95]) per 0.20-g/kg/d increment (*P* = .34 for difference in coefficients) (Table 2). The association of plant protein was similar in the participants younger than 75 years (HR, 0.78 [95% CI, 0.57-1.07]) vs 75 years or older (0.81 [95% CI, 0.65-1.00]) per 0.20-g/kg/d increment in plant protein intake (*P* for interaction = .82). The association was also similar for animal protein intake for those younger than 75 years (HR, 0.93 [95% CI, 0.82-1.06]) and 75 years or older (HR, 0.87 [95% CI, 0.80-0.95]) per 0.20-g/kg/d increment (*P* for interaction = .32) (Table 3). Associations with mortality followed a linear trend except for plant protein intake in the participants younger than 75 years (nadir at 0.45-0.50 g/kg/d; *P* for nonlinearity = .02) (Figure 2).

### Interactions and Ancillary Analyses

The inverse association between total protein intake and mortality was stronger among participants without CKD (HR, 0.85 [95% CI, 0.79-0.92]) per 0.20-g/kg/d increment) relative to those with CKD (HR, 0.92 [95% CI, 0.86-0.98] per 0.20-g/kg/d increment; *P* for interaction = .02) (Table 2). Plant protein intake was more strongly associated with lower mortality in participants without CKD (HR, 0.61 [95% CI, 0.50-0.76]) than with CKD (HR, 0.80 [95% CI, 0.65-0.98]; *P* = .005 for interaction), while risk of mortality was similar in persons with animal protein intake without and with CKD (HRs, 0.89 [95% CI, 0.82-0.98] and 0.88 [95% CI, 0.81-0.95]; *P* for interaction = .74) (Table 2). No significant interactions between total protein intake and CKD arose in any individual cohort or among men or women (eFigures 2 and 3 in Supplement 1).

When examining the main animal and plant protein sources among participants with CKD, fish and cereal protein intake showed inverse associations with mortality (HRs, 0.90 [95% CI, 0.84-0.97] and 0.84 [95% CI, 0.72-0.97] per 0.2-g/kg/d increment, respectively), while dairy and meat protein displayed nonsignificant trends (HRs, 0.95 [95% CI, 0.89-1.00] for dairy protein and 0.96 [95% CI, 0.91-1.02] for meat protein) (eTable 4 in Supplement 1). Increasing plant protein intake over time (but not total or animal protein) was associated with lower mortality (eTable 5 in Supplement 1) even though the proportion of plant protein was not associated with mortality risk (eFigure 4 in Supplement 1). Study associations remained similar or increased in 11 of the 15 sensitivity analyses (eTable 6 in Supplement 1).

## Discussion

### Interpretation

Results of this multicohort study are in line with those of observational studies^10,11,12^ that have found neutral or inverse associations between protein intake and mortality among older persons with CKD stages 3 to 5 not receiving dialysis. First, in 3892 middle aged and older Korean adults, higher total protein intake showed a null association with 11-year all-cause mortality.^10^ Second, among 356 French patients with CKD over 60 years, higher total protein intake was not associated with increased mortality after 3 years.^12^ Third, in 259 Japanese adults with CKD and older than 65 years, higher total protein intake was associated with lower risk of all-cause death over 4 years, although participants had been advised to limit protein intake depending on their CKD stage.^11^

The latest Cochrane systematic reviews^13,14^ of randomized clinical trials found that among adults of all ages without diabetes and with CKD, protein intake of either 0.30 to 0.40 or 0.50 to 0.60 g/kg/d probably does not influence the risk of death when compared with 0.80 g/kg/d or greater, while in adults with diabetic kidney disease, protein intake of 0.60 to 0.80 g/kg/d may make little difference in the risk of mortality when compared with 1.00 g/kg/d or greater.

In our analyses, we observed an inverse association between total protein intake and mortality among participants with CKD but a somewhat weaker one than among those without CKD. Together with the previous studies, this suggests that the benefits of proteins may outweigh the risks in older adults with mild or moderate CKD, in whom disease progression may play a more limited role in survival.

Specifically, protein deficiency in older adults may cause impairments of muscular, skeletal, and immune function, while higher protein intake has been associated with increased muscle mass and strength, slower rate of bone loss, higher bone mineral density, lower risk of frailty, and improved cardiovascular function and recovery from illness (including wound healing).^1,2^ Protein supplementation appears to reduce the risk of death in older persons (possibly by elevating branched-chained amino acid levels), especially in older patients and in the presence of malnutrition or other geriatric syndromes.^1,2,28,29^

Evidence linking protein intake to CKD progression in older adults exists as well, although it is not as consistent. In 3 cohort studies totaling more than 2700 participants,^11,30,31^ one report found slower eGFR decline and another found faster eGFR decline but similar risk of end-stage kidney disease linked to higher protein intake. In the same cohorts, analyses of protein sources showed that plant protein was either not associated with eGFR changes or associated with a slower decline in kidney function.^30,31^ Randomized clinical trials indicate that, among adults with diabetic kidney disease of all ages, protein intake of 0.60 to 0.80 g/kg/d has uncertain effects on changes in eGFR compared with intake of 1.00 g/kg/d or greater.^14^

In our study, the inverse association between plant protein intake and mortality was substantially weaker among the participants with CKD than among those without CKD. Any explanation for the observed differences must be conjectural. On the one hand, the somewhat lower biological value of plant protein could moderate the beneficial actions of protein on muscle mass and function, which could differentially affect older adults with and without CKD (in our study, participants with CKD were more likely to be frail).^15,32^ On the other hand, plants are a source of phosphorus and potassium, which could increase the risk of hyperphosphatemia and hyperkalemia in persons with CKD, particularly at stages 3B and higher.^33^ In any case, since we found comparable associations of plant and animal protein intake with mortality among participants with CKD, it cannot be inferred that plant protein intake should be discouraged.

### Generalizability

The biological actions of protein sources could depend on total protein intake and the proportion of plant protein in the diet. Not only did 68% of total protein come from animal sources in our study, but the mean (SD) protein intake was well above the current recommendations for persons with moderate CKD.^5,6^ This may impair the generalizability of our findings to older adults following plant-based and/or low protein diets, and it is uncertain whether these results could be applied to persons with severe CKD. The generalizability of findings from the SNAC-K and Seniors-ENRICA 2 cohorts to the general population of such countries may be limited. Finally, participants in the Seniors-ENRICA 1 cohort were largely White (99.3%) and we lacked data on race and ethnicity in Seniors-ENRICA 2 and SNAC-K.

### Limitations

This study has limitations. The instruments used to estimate nutrient intake in SNAC-K and Seniors-ENRICA had fundamental differences and have shown moderate reproducibility and validity.^21,22^ Although we used as many food records as possible to minimize measurement error, longitudinal dietary information was not available in all participants, and we did not know whether participants with CKD had been set a protein intake target by health care professionals. As in any observational study, we could not entirely disentangle protein intake from other nutrients, and there is potential for residual confounding, as many variables were self-reported, while some potential confounders could not be accounted for.

Among other study limitations, measured GFR was not available in any cohort. Estimated GFR could be subject to some measurement error, even more so when we used an equation based on creatinine levels alone.^23,34^ Moreover, information on CKD and potential confounders was not obtained from the same sources at every wave and cohort. Since most CKD cases were ascertained through eGFR and not medical records, we could not differentiate between CKD causes and transient and chronic declines in kidney function.

Several of these limitations were addressed in sensitivity analyses. Although the inverse associations of protein intake with mortality in participants with CKD were sometimes lost when making alternative assumptions, at no instance did a direct association appear.

## Conclusions

In this multicohort study, higher total, animal, and plant protein intake were associated with lower mortality in older adults with CKD. The stronger associations in participants without CKD suggest that the benefits of proteins may outweigh the downsides in older persons with mild or moderate CKD.

It is uncertain whether our findings apply to settings where protein intake is lower or plant foods are the main protein source. Studies in older adults with severe CKD, as well as those from ethnically diverse populations, are also needed. Whether modification of protein intake has effects on mortality in older persons with CKD may be investigated in future randomized trials.

## Notes

Supplement 1. **eMethods 1**. Potential Confounders **eMethods 2.** Multiple Imputation by Chained Equations **eMethods 3**. Sensitivity Analyses **eTable 1.** Participant Observations With Missing Data for Each Variable, Time Point, and Cohort **eTable 2.** Characteristics of the Participant Observations, Stratified by Chronic Kidney Disease and Age **eTable 3.** Characteristics of the Participant Observations, Stratified by Chronic Kidney Disease and Cohort **eTable 4.** Hazard Ratios (95% CIs) of the Associations of the Main Sources of Animal and Plant Protein Intake With 10-Year All-Cause Mortality, Stratified by Chronic Kidney Disease **eTable 5.** Hazard Ratios (95% CIs) of the Associations of Changes in Total, Animal, and Plant Protein Intake With 10-Year All-Cause Mortality, Stratified by Chronic Kidney Disease **eTable 6.** Sensitivity Analyses: Hazard Ratios (95% CIs) of the Associations of Total, Animal, and Plant Protein Intake With 10-Year All-Cause Mortality, Stratified by Chronic Kidney Disease **eFigure 1.** Participants’ Flowchart, Stratified by Cohort **eFigure 2.** Association of Total Protein Intake With 10-Year All-Cause Mortality, Stratified by Chronic Kidney Disease and Cohort **eFigure 3.** Association of Total Protein Intake With 10-Year All-Cause Mortality, Stratified by Chronic Kidney Disease and Sex **eFigure 4.** Association of the Proportion of Plant Protein With 10-Year All-Cause Mortality, Stratified by Chronic Kidney Disease and Age
eReferences.

Supplement 2. Data Sharing Statement
