# Supplementary material for: Protein Intake and Mortality in Older Adults With Chronic Kidney Disease
Source: JAMA Netw Open. 2024 Aug 7;7(8):e2426577. doi: 10.1001/jamanetworkopen.2024.26577 (PMC11307132; doi:10.1001/jamanetworkopen.2024.26577)
Supplement: Supplement 1. — eMethods 1. Potential Confounders eMethods 2. Multiple Imputation by Chained Equations eMethods 3. Sensitivity Analyses eTable 1. Participant Observations With Missing Data for Each Variable, Time Point, and Cohort eTable 2. Characteristics of the Participant Observations, Stratified by Chronic Kidney Disease and Age eTable 3. Characteristics of the Participant Observations, Stratified by Chronic Kidney Disease and Cohort eTable 4. Hazard Ratios (95% CIs) of the Associations of the Main Sources of Animal and Plant Protein Intake With 10-Year All-Cause Mortality, Stratified by Chronic Kidney Disease eTable 5. Hazard Ratios (95% CIs) of the Associations of Changes in Total, Animal, and Plant Protein Intake With 10-Year All-Cause Mortality, Stratified by Chronic Kidney Disease eTable 6. Sensitivity Analyses: Hazard Ratios (95% CIs) of the Associations of Total, Animal, and Plant Protein Intake With 10-Year All-Cause Mortality, Stratified by Chronic Kidney Disease eFigure 1. Participants’ Flowchart, Stratified by Cohort eFigure 2. Association of Total Protein Intake With 10-Year All-Cause Mortality, Stratified by Chronic Kidney Disease and Cohort eFigure 3. Association of Total Protein Intake With 10-Year All-Cause Mortality, Stratified by Chronic Kidney Disease and Sex eFigure 4. Association of the Proportion of Plant Protein With 10-Year All-Cause Mortality, Stratified by Chronic Kidney Disease and Age eReferences. [file jamanetwopen-e2426577-s001.pdf]

Supplementary Online Content

Carballo-Casla A, Avesani CM, Beridze G, et al. Protein intake and mortality in older adults with chronic kidney disease. *JAMA Netw Open*. 2024;7(8):e2426577. doi:10.1001/jamanetworkopen.2024.26577

eMethods 1. Potential Confounders

eMethods 2. Multiple Imputation by Chained Equations

eMethods 3. Sensitivity Analyses

eTable 1. Participant Observations With Missing Data for Each Variable, Collection Wave, and Cohort

eTable 2. Characteristics of the Participant Observations, Stratified by Chronic Kidney Disease and Age

eTable 3. Characteristics of the Participant Observations, Stratified by Chronic Kidney Disease and Cohort

eTable 4. Hazard Ratios (95% CIs) of the Associations of the Main Sources of Animal and Plant Protein Intake With 10-Year All-Cause Mortality, Stratified by Chronic Kidney Disease

eTable 5. Hazard Ratios (95% CIs) of the Associations of Changes in Total, Animal, and Plant Protein Intake With 10-Year All-Cause Mortality, Stratified by Chronic Kidney Disease

eTable 6. Sensitivity Analyses: Hazard Ratios (95% CIs) of the Associations of Total, Animal, and Plant Protein Intake With 10-Year All-Cause Mortality, Stratified by Chronic Kidney Disease

eFigure 1. Participants' Flowchart, Stratified by Cohort

eFigure 2. Association of Total Protein Intake With 10-Year All-Cause Mortality, Stratified by Chronic Kidney Disease and Cohort

eFigure 3. Association of Total Protein Intake With 10-Year All-Cause Mortality, Stratified by Chronic Kidney Disease and Sex

eFigure 4. Association of the Proportion of Plant Protein With 10-Year All-Cause Mortality, Stratified by Chronic Kidney Disease and Age

eReferences.

This supplementary material has been provided by the authors to give readers additional information about their work.

© 2024 Carballo-Casla A et al. *JAMA Network Open*.

eMethods 1. Potential Confounders

Sociodemographic and lifestyle variables were generally self-reported, although physical activity was also estimated with accelerometers in Seniors-ENRICA 2. Weight and height were measured in standardized conditions, and self-reported data were used when they were not. Nutrient intakes were estimated from dietary histories and food frequency questionnaires (FFQs).[2–5]

In SNAC-K, information on morbidities was based on clinical examinations, blood tests, medications, patient histories, and inpatient and outpatient medical records.[1] In Seniors-ENRICA 1, medical diagnoses were self-reported save for diabetes and depression and mood disorders, where data on blood glucose levels and medications were also used. Operationalization of morbidities was similar in Seniors-ENRICA 2, except that primary care records were available for consultation.

© 2024 Carballo-Casla A et al. *JAMA Network Open*.

eMethods 2. Multiple Imputation by Chained Equations

Prior to the estimation of energy and nutrient intakes, missing FFQ items were imputed in SNAC-K participants who completed ≥50% of the questions (note that Seniors-ENRICA's dietary histories always provide complete records).[6]

Missing data on estimated glomerular filtration rates (eGFR) and potential confounders were imputed in all cohorts, as were energy and nutrient intakes in probable dietary misreporters (≤800 or ≥5000 kilocalories/day in men and ≤500 or ≥4000 kilocalories/day in women). Continuous variables were imputed using predictive mean matching, and categorical variables using ordered logistic, multinomial logistic, or logistic regression, as appropriate. Nonmissing potential confounders were entered in the equations to improve predictions.

Our analyses used missing at random assumptions: we ran estimation commands in the imputed data and adjusted coefficients and standard errors for the variability between the 10 imputations according to the combination rules by Rubin.[7]

© 2024 Carballo-Casla A et al. *JAMA Network Open*.

eMethods 3. Sensitivity Analyses

Since data on diet and many potential confounders were self-reported, we excluded the participants with probable cognitive impairment (Mini-Mental State Examination score <24 points) at baseline and stopped updating information on all study variables when such condition presented in the follow-up waves.

Given that the participants with incident chronic kidney disease (CKD) contributed to both the CKD and no CKD risk sets, we restarted the cumulative average of dietary variables and continuous potential confounders at the follow-up wave in which CKD was ascertained.

To minimize the potential for reverse causation (i.e., health status leading to lower protein intake), we omitted the first year of follow-up, expressed protein intake in relative as opposed to absolute terms (percentage of energy intake) and in g/kg of ideal body weight/day (the weight at which body mass index equals 23 kg/m^2), and excluded the observations carrying a medical diagnosis of CKD.

In addition, we excluded the participants who changed their total protein intake ≥50% between the first two food records and excluded the current and remaining observations when such change occurred later in the follow-up.

We alternately adjusted the analyses for the frailty phenotype (unintentional weight loss, exhaustion, weakness, slow walking speed, and low physical activity)[8] and hypertension (systolic blood pressure ≥140 millimeters of mercury, diastolic blood pressure ≥90 millimeters of mercury, use of antihypertensive medication, or medical diagnosis) to reduce residual confounding.

Since information on study variables could not be updated at every wave and cohort, we ran the analyses using baseline data only, with and without coarsened exact matching (a method for improving the estimation of causal effects by reducing imbalance in covariates between participants with and without CKD).[9]

To explore the differences between the Berlin Initiative Study (BIS) equation and others and to reduce potential eGFR misclassification, we alternatively used the Chronic Kidney Disease

© 2024 Carballo-Casla A et al. *JAMA Network Open*.

Epidemiology Collaboration (CKD-EPI) and the BIS2 equation whenever possible (note that data on cystatin-C were only available in SNAC-K and only at baseline).[5]

Since missing at random assumptions may not be verified, we conducted a complete case analysis by excluding the participants without information on every study variable and probable dietary misreporters, and assuming that missing FFQ items represented zero consumption.

Finally, to estimate the average of different study-specific effect sizes, we ran the analysis independently in each cohort and pooled the results using fixed-effects meta-analysis with the inverse-variance estimation method.

© 2024 Carballo-Casla A et al. *JAMA Network Open*.

eTable 1. Participant Observations With Missing Data for Each Variable, Time Point, and Cohort

	SNAC-K			Seniors-ENRICA 1				Seniors-ENRICA 2		
	**Baseline**	**Follow-up 1**	**Follow-up 2**	**Baseline**	**Follow-up 1**	**Follow-up 2**	**Follow-up 3**	**Baseline**	**Follow-up 1**	**Follow-up 2**
Observations	2463	494	1503	3291	2073	869	869	2789	917	917
Dietary variables										
Electronic dietary history	NA	NA	NA	13 (0.40)	4 (0.19)	NA	0 (0)	5 (0.18)	NA	0 (0)
Semi-quantitative FFQ	28 (1.14)	13 (2.63)	18 (1.20)	NA	NA	NA	NA	NA	NA	NA
Chronic kidney disease										
Estimated glomerular filtration rate	56 (2.27)	12 (2.43)	24 (1.60)	20 (0.61)	NA	36 (4.14)	NA	233 (8.35)	93 (10.1)	100 (10.9)
Urine albumin	NA	NA	NA	42 (1.28)	NA	NA	NA	1580 (56.7)	NA	NA
Patient histories	0 (0)	0 (0)	0 (0)	NA	NA	NA	NA	NA	NA	NA
Hospital records	45 (1.83)	2 (0.40)	18 (1.20)	NA	NA	NA	NA	NA	NA	NA
Primary care records	NA	NA	NA	NA	NA	NA	NA	0 (0)	0 (0)	0 (0)
Death certificates	0 (0)	0 (0)	0 (0)	0 (0)	NA	0 (0)	NA	NA	NA	NA
Potential confounders										
Sex	0 (0)	NA	NA	0 (0)	NA	NA	NA	0 (0)	NA	NA
Age	0 (0)	0 (0)	0 (0)	0 (0)	0 (0)	NA	0 (0)	0 (0)	NA	0 (0)
Living arrangement	6 (0.24)	4 (0.81)	8 (0.53)	0 (0)	0 (0)	NA	0 (0)	1 (0.036)	NA	15 (1.64)
Previous occupation	7 (0.28)	NA	NA	287 (8.72)	NA	NA	NA	10 (0.36)	NA	NA
Educational level	3 (0.12)	NA	NA	6 (0.18)	NA	NA	NA	2 (0.072)	NA	NA
Tobacco smoking	40 (1.62)	0 (0)	7 (0.47)	7 (0.21)	0 (0)	NA	0 (0)	0 (0)	NA	0 (0)
Light physical activity	43 (1.75)	84 (17.0)	68 (4.52)	0 (0)	0 (0)	NA	0 (0)	0 (0)	NA	0 (0)
Moderate-to-vigorous physical activity	57 (2.31)	82 (16.6)	70 (4.66)	0 (0)	0 (0)	NA	0 (0)	0 (0)	NA	0 (0)
Body mass index	30 (1.22)	20 (4.05)	19 (1.26)	39 (1.19)	0 (0)	NA	0 (0)	11 (0.39)	NA	14 (1.53)
Morbidities	0 (0)	0 (0)	0 (0)	0 (0)	0 (0)	NA	0 (0)	0 (0)	NA	0 (0)

Values are observations (%). NA: not applicable.

© 2024 Carballo-Casla A et al. *JAMA Network Open*.

Note that data on chronic kidney disease from Seniors-ENRICA 1's follow-up 2 were only used if study participants underwent dietary assessment at follow-up 3, while data on chronic kidney disease from Seniors-ENRICA 2's follow-up 1 were only used if study participants underwent dietary assessment at follow-up 2.

© 2024 Carballo-Casla A et al. *JAMA Network Open*.

eTable 2. Characteristics of the Participant Observations, Stratified by Chronic Kidney Disease and Age

	Chronic kidney disease		No chronic kidney disease	
	**<75 years**	**≥75 years**	**<75 years**	**≥75 years**
Observations	1622	3167	7734	1876
Sociodemographic variables				
Sex − female	790 (48.7)	1936 (61.1)	4175 (54.0)	1083 (57.7)
Age, mean (standard deviation)	69.9 (3.68)	82.1 (4.58)	67.9 (4.11)	78.7 (2.84)
Living arrangement − alone	1253 (77.3)	1666 (52.6)	5913 (76.5)	1219 (65.0)
Previous occupation – manual worker	698 (43.0)	1215 (38.4)	3040 (39.3)	894 (47.6)
Educational level				
Primary or less	696 (42.9)	1302 (41.1)	3197 (41.3)	1071 (57.1)
Secondary	486 (30.0)	1163 (36.7)	2299 (29.7)	438 (23.3)
University	440 (27.1)	703 (22.2)	2238 (28.9)	367 (19.6)
Lifestyle variables				
Tobacco smoking				
Never	761 (46.9)	1813 (57.2)	3743 (48.4)	1117 (59.5)
Former	644 (39.7)	1166 (36.8)	3032 (39.2)	627 (33.4)
Current	218 (13.4)	188 (5.93)	959 (12.4)	132 (7.01)
Light physical activity				
Never	117 (7.24)	333 (10.5)	277 (3.58)	109 (5.79)
Less than monthly	111 (6.85)	308 (9.71)	352 (4.54)	120 (6.38)
Monthly	383 (23.6)	555 (17.5)	1483 (19.2)	404 (21.5)
Weekly	656 (40.5)	1133 (35.8)	3652 (47.2)	838 (44.7)
Daily	354 (21.8)	839 (26.5)	1971 (25.5)	406 (21.6)
Moderate-to-vigorous physical activity				
Never	676 (41.7)	1856 (58.6)	2461 (31.8)	842 (44.9)
Less than monthly	283 (17.5)	512 (16.2)	1303 (16.8)	342 (18.2)
Monthly	316 (19.5)	357 (11.3)	1736 (22.4)	351 (18.7)
Weekly	273 (16.9)	326 (10.3)	1826 (23.6)	251 (13.4)
Daily	73 (4.53)	117 (3.69)	409 (5.28)	91 (4.85)
Body mass index, mean (standard deviation)	28.5 (4.80)	26.9 (4.34)	27.5 (4.23)	27.3 (4.28)
Morbidity variables				
Chronic kidney disease				
Stage 1	36 (2.22)	13 (0.41)	NA	NA
Stage 2	489 (30.1)	237 (7.48)	NA	NA
Stage 3A	1030 (63.5)	2293 (72.4)	NA	NA
Stage 3B	67 (4.13)	624 (19.7)	NA	NA
Diabetes	385 (23.7)	612 (19.3)	1161 (15.0)	378 (20.1)
Cardiovascular disease	241 (14.9)	916 (28.9)	682 (8.82)	338 (18.0)
Chronic lung disease	178 (11.0)	428 (13.5)	862 (11.1)	297 (15.8)
Musculoskeletal disease	713 (44.0)	1563 (49.4)	3502 (45.3)	1219 (65.0)
Cancer	169 (10.4)	478 (15.1)	595 (7.69)	233 (12.4)
Depression and mood disorders	195 (12.0)	325 (10.3)	798 (10.3)	239 (12.7)

© 2024 Carballo-Casla A et al. *JAMA Network Open*.

Dietary variables, mean (standard deviation)				
Energy (g/kg/day)	27.1 (7.62)	28.4 (8.75)	27.3 (7.35)	27.8 (6.90)
Total protein (g/kg/day)	1.18 (0.39)	1.13 (0.36)	1.17 (0.35)	1.20 (0.32)
Animal protein (g/kg/day)	0.80 (0.32)	0.77 (0.27)	0.79 (0.27)	0.79 (0.24)
Plant protein (g/kg/day)	0.38 (0.14)	0.36 (0.13)	0.39 (0.14)	0.41 (0.13)
Monounsaturated fat (g/kg/day)	0.47 (0.18)	0.47 (0.17)	0.47 (0.17)	0.48 (0.16)
Sugar (g/kg/day)	1.21 (0.48)	1.35 (0.56)	1.24 (0.48)	1.32 (0.49)
Alcohol (g/kg/day)	0.13 (0.19)	0.11 (0.14)	0.14 (0.18)	0.11 (0.15)
Sodium (mg/kg/day)	36.7 (13.9)	38.0 (14.8)	36.5 (13.4)	37.4 (12.3)

Values are observations (%) unless otherwise indicated. NA: not applicable.

© 2024 Carballo-Casla A et al. *JAMA Network Open*.

eTable 3. Characteristics of the Participant Observations, Stratified by Chronic Kidney Disease and Cohort

	Chronic kidney disease			No chronic kidney disease		
	**SNAC-K**	**Seniors-ENRICA 1**	**Seniors-ENRICA 2**	**SNAC-K**	**Seniors-ENRICA 1**	**Seniors-ENRICA 2**
Observations	1973	1885	931	2487	4348	2775
Sociodemographic variables						
Sex − female	1368 (69.3)	924 (49.0)	434 (46.6)	1395 (56.1)	2374 (54.6)	1489 (53.7)
Age, mean (standard deviation)	81.1 (6.89)	75.5 (7.28)	76.6 (4.92)	68.0 (6.42)	69.6 (5.67)	72.4 (4.41)
Living arrangement − alone	787 (39.9)	1464 (77.7)	669 (71.8)	1392 (56.0)	3594 (82.7)	2146 (77.3)
Previous occupation – manual worker	469 (23.8)	992 (52.6)	452 (48.5)	378 (15.2)	2185 (50.3)	1371 (49.4)
Educational level						
Primary or less	339 (17.2)	1100 (58.3)	560 (60.2)	212 (8.52)	2316 (53.3)	1741 (62.7)
Secondary	1076 (54.5)	413 (21.9)	160 (17.2)	1109 (44.6)	1105 (25.4)	523 (18.9)
University	559 (28.3)	373 (19.8)	211 (22.7)	1166 (46.9)	928 (21.3)	511 (18.4)
Lifestyle variables						
Tobacco smoking						
Never	1081 (54.8)	1031 (54.7)	462 (49.6)	973 (39.1)	2456 (56.5)	1432 (51.6)
Former	733 (37.2)	675 (35.8)	402 (43.2)	1132 (45.5)	1445 (33.2)	1082 (39.0)
Current	159 (8.07)	179 (9.51)	67 (7.20)	382 (15.4)	447 (10.3)	261 (9.41)
Light physical activity						
Never	198 (10.0)	229 (12.1)	24 (2.58)	114 (4.60)	247 (5.68)	24 (0.86)
Less than monthly	196 (9.92)	175 (9.28)	48 (5.16)	115 (4.63)	292 (6.72)	64 (2.31)
Monthly	155 (7.85)	495 (26.3)	288 (30.9)	241 (9.68)	1047 (24.1)	599 (21.6)
Weekly	679 (34.4)	659 (35.0)	451 (48.4)	1033 (41.5)	1651 (38.0)	1806 (65.1)
Daily	746 (37.8)	327 (17.3)	120 (12.9)	984 (39.5)	1111 (25.6)	282 (10.2)

© 2024 Carballo-Casla A et al. *JAMA Network Open*.

Moderate-to-vigorous physical activity						
Never	1059 (53.7)	1165 (61.8)	308 (33.1)	687 (27.6)	2168 (49.9)	448 (16.1)
Less than monthly	370 (18.8)	255 (13.5)	170 (18.3)	531 (21.3)	730 (16.8)	384 (13.8)
Monthly	187 (9.47)	263 (14.0)	223 (24.0)	449 (18.1)	814 (18.7)	823 (29.7)
Weekly	263 (13.3)	165 (8.75)	171 (18.4)	649 (26.1)	528 (12.1)	900 (32.4)
Daily	94 (4.78)	37 (1.96)	59 (6.34)	172 (6.90)	108 (2.48)	220 (7.93)
Body mass index, mean (standard deviation)	25.8 (4.00)	28.8 (4.58)	28.2 (4.62)	25.9 (3.71)	28.3 (4.21)	27.6 (4.35)
Morbidity variables						
Chronic kidney disease						
Stage 1	3 (0.15)	26 (1.38)	26 (2.15)	NA	NA	NA
Stage 2	6 (0.30)	524 (27.8)	524 (21.1)	NA	NA	NA
Stage 3A	1520 (77.0)	1178 (62.5)	1178 (67.1)	NA	NA	NA
Stage 3B	444 (22.5)	157 (8.33)	157 (9.67)	NA	NA	NA
Diabetes	243 (12.3)	483 (25.6)	271 (29.1)	207 (8.32)	775 (17.8)	557 (20.1)
Cardiovascular disease	671 (34.0)	235 (12.5)	251 (27.0)	331 (13.3)	292 (6.72)	397 (14.3)
Chronic lung disease	244 (12.4)	197 (10.5)	165 (17.7)	276 (11.1)	473 (10.9)	410 (14.8)
Musculoskeletal disease	600 (30.4)	1083 (57.5)	593 (63.7)	588 (23.6)	2457 (56.5)	1676 (60.4)
Cancer	323 (16.4)	94 (4.99)	230 (24.7)	248 (9.97)	162 (3.73)	418 (15.1)
Depression and mood disorders	189 (9.58)	226 (12.0)	105 (11.3)	217 (8.73)	494 (11.4)	326 (11.7)
Dietary variables, mean (standard deviation)						
Energy (g/kg/day)	29.3 (10.4)	27.1 (7.19)	26.9 (4.90)	26.5 (9.18)	27.8 (7.08)	27.7 (5.26)
Total protein (g/kg/day)	1.01 (0.38)	1.24 (0.38)	1.22 (0.23)	0.93 (0.34)	1.26 (0.32)	1.27 (0.26)
Animal protein (g/kg/day)	0.71 (0.28)	0.83 (0.32)	0.80 (0.19)	0.65 (0.26)	0.84 (0.26)	0.84 (0.22)
Plant protein (g/kg/day)	0.30 (0.12)	0.41 (0.13)	0.42 (0.10)	0.28 (0.11)	0.43 (0.14)	0.43 (0.11)
Monounsaturated fat (g/kg/day)	0.45 (0.19)	0.47 (0.17)	0.49 (0.13)	0.42 (0.17)	0.49 (0.17)	0.51 (0.15)
Sugar (g/kg/day)	1.41 (0.63)	1.25 (0.47)	1.20 (0.40)	1.23 (0.58)	1.28 (0.47)	1.25 (0.42)
Alcohol (g/kg/day)	0.11 (0.11)	0.12 (0.20)	0.11 (0.15)	0.14 (0.12)	0.13 (0.20)	0.12 (0.17)
Sodium (mg/kg/day)	38.8 (16.3)	37.1 (14.4)	35.9 (9.74)	34.8 (14.2)	37.6 (14.1)	36.8 (10.5)

Values are observations (%) unless otherwise indicated. NA: not applicable.

© 2024 Carballo-Casla A et al. *JAMA Network Open*.

eTable 4. Hazard Ratios (95% CIs) of the Associations of the Main Sources of Animal and Plant Protein Intake With 10-Year All-Cause Mortality, Stratified by Chronic Kidney Disease

	**Chronic kidney disease**	**No chronic kidney disease**
Deaths/observations	838/4789	630/9610
**Dairy protein**		
0.10 g/kg/day	Reference	Reference
0.20 g/kg/day	0.82 (0.72-0.95)	1.01 (0.87-1.17)
0.30 g/kg/day	0.74 (0.60-0.91)	1.01 (0.82-1.25)
0.40 g/kg/day	0.71 (0.57-0.89)	1.01 (0.81-1.27)
0.50 g/kg/day	0.69 (0.54-0.89)	1.01 (0.78-1.31)
Per 0.10 g/kg/day	0.95 (0.89-1.00)	1.01 (0.95-1.08)
**Meat protein**		
0.10 g/kg/day	Reference	Reference
0.20 g/kg/day	0.87 (0.77-0.97)	0.83 (0.73-0.95)
0.30 g/kg/day	0.78 (0.64-0.95)	0.72 (0.57-0.90)
0.40 g/kg/day	0.76 (0.61-0.94)	0.66 (0.51-0.85)
0.50 g/kg/day	0.76 (0.60-0.96)	0.63 (0.48-0.82)
Per 0.10 g/kg/day	0.96 (0.91-1.02)	0.91 (0.86-0.97)
**Fish protein**		
0.10 g/kg/day	Reference	Reference
0.15 g/kg/day	0.91 (0.85-0.98)	0.91 (0.84-1.00)
0.20 g/kg/day	0.85 (0.77-0.95)	0.85 (0.75-0.97)
0.25 g/kg/day	0.82 (0.72-0.92)	0.81 (0.70-0.94)
0.30 g/kg/day	0.79 (0.69-0.91)	0.78 (0.66-0.93)
Per 0.10 g/kg/day	0.90 (0.84-0.97)	0.89 (0.81-0.97)
**Cereal protein**		
0.10 g/kg/day	Reference	Reference
0.15 g/kg/day	0.90 (0.79-1.01)	0.80 (0.71-0.90)
0.20 g/kg/day	0.80 (0.65-0.99)	0.66 (0.54-0.82)
0.25 g/kg/day	0.72 (0.56-0.94)	0.59 (0.45-0.77)
0.30 g/kg/day	0.65 (0.48-0.89)	0.55 (0.41-0.74)
Per 0.10 g/kg/day	0.84 (0.72-0.97)	0.80 (0.68-0.93)

Cox proportional hazards regression models. Protein intake was modelled as a continuous variable (per 0.10 g/kg/day) or a 3-knot restricted cubic spline otherwise. Hazard ratios (95% confidence intervals) of the associations of dairy, meat, and fish protein intake were obtained from models integrating interaction terms between the relevant source of animal protein and chronic kidney

© 2024 Carballo-Casla A et al. *JAMA Network Open*.

disease, between other animal proteins and chronic kidney disease, and between plant protein and chronic kidney disease. Hazard ratios (95% confidence intervals) of the associations of cereal protein intake were obtained from models integrating interaction terms between cereal protein and chronic kidney disease, between other plant proteins and chronic kidney disease, and between animal protein and chronic kidney disease.

Models were adjusted for cohort (SNAC-K, Seniors-ENRICA 1, or Seniors-ENRICA 2), sex (male or female), age (years), living arrangement (alone or with spouse/partner/other), previous occupation (manual worker or non-manual worker), educational level (primary or less, secondary, or university), tobacco smoking (never, former, or current), light physical activity (daily, weekly, monthly, less often, or never), moderate-to-vigorous physical activity (daily, weekly, monthly, less often, or never), body mass index (kg/m^2), diabetes, cardiovascular disease (ischemic heart disease, cerebrovascular disease, or heart failure), chronic lung disease (chronic obstructive pulmonary disease/emphysema/chronic bronchitis or asthma), musculoskeletal disease (osteoarthritis and other degenerative joint diseases, inflammatory arthropathies, or current pathological fracture), cancer (hematological and solid neoplasms), depression and mood disorders (e.g., manic episode, bipolar affective disorder), energy intake (g/kg/day), monounsaturated fat (g/kg/day), sugar (g/kg/day), alcohol (g/kg/day), and sodium intake (mg/kg/day).

© 2024 Carballo-Casla A et al. *JAMA Network Open*.

eTable 5. Hazard Ratios (95% CIs) of the Associations of Changes in Total, Animal, and Plant Protein Intake With 10-Year All-Cause Mortality, Stratified by Chronic Kidney Disease

	**Chronic kidney disease**	**No chronic kidney disease**
Deaths/observations	302/1368	194/3323
**Total protein**		
-0.20 g/kg/day	Reference	Reference
-0.10 g/kg/day	0.96 (0.89-1.03)	0.92 (0.85-0.99)
0.00 g/kg/day	0.91 (0.80-1.05)	0.86 (0.74-0.99)
+0.10 g/kg/day	0.87 (0.71-1.07)	0.81 (0.66-1.00)
+0.20 g/kg/day	0.83 (0.63-1.09)	0.78 (0.60-1.03)
Per +0.10 g/kg/day	0.96 (0.90-1.03)	0.95 (0.89-1.02)
**Animal protein**		
-0.20 g/kg/day	Reference	Reference
-0.10 g/kg/day	0.95 (0.88-1.03)	0.90 (0.83-0.98)
0.00 g/kg/day	0.89 (0.77-1.03)	0.83 (0.71-0.97)
+0.10 g/kg/day	0.82 (0.66-1.02)	0.78 (0.63-0.98)
+0.20 g/kg/day	0.75 (0.56-1.00)	0.76 (0.57-1.00)
Per +0.10 g/kg/day	0.96 (0.90-1.03)	0.95 (0.89-1.02)
**Plant protein**		
-0.10 g/kg/day	Reference	Reference
-0.05 g/kg/day	0.84 (0.76-0.94)	0.83 (0.74-0.94)
0.00 g/kg/day	0.72 (0.60-0.88)	0.70 (0.56-0.87)
+0.05 g/kg/day	0.64 (0.48-0.84)	0.60 (0.44-0.81)
+0.10 g/kg/day	0.57 (0.39-0.83)	0.52 (0.35-0.76)
Per +0.10 g/kg/day	0.81 (0.68-0.97)	0.79 (0.66-0.94)

* Two-tailed p value<0.05.

Cox proportional hazards regression models. Changes in protein intake were modelled as a continuous variable (per +0.10 g/kg/day) or a 3-knot restricted cubic spline otherwise. Hazard ratios (95% confidence intervals) were obtained from models with interaction terms between changes in protein intake and baseline chronic kidney disease. Models on protein sources integrated multiplicative interactions between changes in animal protein and baseline CKD and between changes in plant protein intake and baseline CKD.

© 2024 Carballo-Casla A et al. *JAMA Network Open*.

Models were adjusted for cohort (SNAC-K, Seniors-ENRICA 1, or Seniors-ENRICA 2), sex (male or female), baseline age (years), living arrangement (remained living alone, started living alone, stopped living alone, or remained with spouse/partner/other), previous occupation (manual worker or non-manual worker), educational level (primary or less, secondary, or university), tobacco smoking (remained a non-smoker, started smoking, stopped smoking, or remained a smoker), baseline light physical activity (daily, weekly, monthly, less often, or never), changes in light physical activity (nine categories, from daily-to-never to never-to-daily), baseline moderate-to-vigorous physical activity (daily, weekly, monthly, less often, or never), changes in moderate-to-vigorous physical activity (nine categories, from daily-to-never to never-to-daily), baseline body mass index (kg/m^2), changes in body mass index (kg/m^2), diabetes (remained free from the disease, disease incidence, or remained with the disease), cardiovascular disease (remained free from the disease, disease incidence, or remained with the disease), chronic lung disease (remained free from the disease, disease incidence, or remained with the disease), musculoskeletal disease (remained free from the disease, disease incidence, disease remission, or remained with the disease), cancer (remained free from the disease, disease incidence, or remained with the disease), depression and mood disorders (remained free from the disease, disease incidence, disease remission, or remained with the disease), baseline energy (g/kg/day), changes in energy intake (g/kg/day), baseline monounsaturated fat (g/kg/day), changes in monounsaturated fat intake (g/kg/day), baseline sugar (g/kg/day), changes in sugar intake (g/kg/day), baseline alcohol (g/kg/day), changes in alcohol intake (g/kg/day), baseline sodium (mg/kg/day), and changes in sodium intake (mg/kg/day). Analyses of changes in total protein intake were adjusted for baseline total protein (g/kg/day). Analyses of changes in animal protein intake were adjusted for baseline animal protein (g/kg/day) and baseline plant protein (g/kg/day). Analyses of changes in plant protein intake were adjusted for baseline plant protein (g/kg/day) and baseline animal protein (g/kg/day).

© 2024 Carballo-Casla A et al. *JAMA Network Open*.

eTable 6. Sensitivity Analyses: Hazard Ratios (95% CIs) of the Associations of Total, Animal, and Plant Protein Intake With 10-Year All-Cause Mortality, Stratified by Chronic Kidney Disease

	Chronic kidney disease	No chronic kidney disease
**Excluding cognitive impairment**		
Deaths/observations	797/4666	612/9484
Total protein	0.93 (0.86-0.99)	0.87 (0.80-0.94)
Animal protein	0.89 (0.82-0.96)	0.91 (0.83-0.99)
Plant protein	0.82 (0.66-1.01)	0.64 (0.51-0.79)
**Cumulative average after incident CKD**		
Deaths/observations	838/4789	630/9610
Total protein	0.91 (0.85-0.98)	0.85 (0.79-0.92)
Animal protein	0.88 (0.81-0.95)	0.90 (0.82-0.98)
Plant protein	0.79 (0.65-0.97)	0.61 (0.50-0.76)
**Omitting the first year of follow-up**		
Deaths/observations	808/4759	613/9593
Total protein	0.93 (0.86-0.99)	0.85 (0.79-0.92)
Animal protein	0.89 (0.82-0.96)	0.89 (0.82-0.97)
Plant protein	0.82 (0.67-1.01)	0.62 (0.50-0.78)
**Protein as % of energy intake**		
Deaths/observations	838/4789	630/9610
Total protein	0.94 (0.89-1.00)	0.92 (0.86-0.97)
Animal protein	0.92 (0.86-0.97)	0.93 (0.88-0.99)
Plant protein	0.87 (0.75-1.02)	0.70 (0.59-0.83)
**Protein as g/kg of ideal body weight/day**		
Deaths/observations	838/4789	630/9610
Total protein	0.93 (0.88-0.99)	0.88 (0.82-0.94)
Animal protein	0.89 (0.83-0.95)	0.91 (0.84-0.98)
Plant protein	0.84 (0.70-1.01)	0.66 (0.54-0.79)
**Excluding large changes in protein intake**		
Deaths/observations	786/4224	596/8776
Total protein	0.93 (0.86-1.00)	0.86 (0.79-0.93)
Animal protein	0.89 (0.82-0.97)	0.91 (0.83-0.99)
Plant protein	0.83 (0.67-1.02)	0.62 (0.50-0.78)
**Adjusting for the frailty phenotype**		
Deaths/observations	838/4789	630/9610
Total protein	0.92 (0.86-0.99)	0.85 (0.79-0.92)
Animal protein	0.89 (0.82-0.96)	0.89 (0.82-0.97)
Plant protein	0.81 (0.66-1.00)	0.62 (0.50-0.77)
**Adjusting for hypertension**		
Deaths/observations	838/4789	630/9610
Total protein	0.92 (0.86-0.98)	0.85 (0.79-0.92)
Animal protein	0.88 (0.81-0.95)	0.89 (0.82-0.98)
Plant protein	0.80 (0.65-0.98)	0.61 (0.49-0.76)

© 2024 Carballo-Casla A et al. *JAMA Network Open*.

**Baseline data only**		
Deaths/observations	816/2547	652/5996
Total protein	0.97 (0.92-1.03)	0.91 (0.85-0.97)
Animal protein	0.95 (0.89-1.01)	0.95 (0.88-1.02)
Plant protein	0.95 (0.79-1.13)	0.74 (0.62-0.89)
**Coarsened exact matching (baseline data)**		
Deaths/observations	594/2168	581/5380
Total protein	0.98 (0.91-1.05)	0.88 (0.81-0.97)
Animal protein	0.93 (0.86-1.01)	0.90 (0.82-0.99)
Plant protein	0.85 (0.67-1.10)	0.63 (0.51-0.79)
**BIS2 equation for eGFR**		
Deaths/observations	707/4158	761/10241
Total protein	0.92 (0.85-0.99)	0.87 (0.81-0.93)
Animal protein	0.87 (0.80-0.94)	0.91 (0.84-0.98)
Plant protein	0.82 (0.66-1.02)	0.61 (0.50-0.75)
**CKD-EPI equation for eGFR**		
Deaths/observations	580/3016	888/11383
Total protein	0.91 (0.84-0.98)	0.88 (0.82-0.95)
Animal protein	0.87 (0.79-0.95)	0.91 (0.84-0.97)
Plant protein	0.81 (0.64-1.02)	0.67 (0.54-0.81)
**Excluding CKD medical diagnoses**		
Deaths/observations	819/4680	630/9610
Total protein	0.92 (0.86-0.99)	0.85 (0.78-0.91)
Animal protein	0.88 (0.82-0.96)	0.89 (0.81-0.97)
Plant protein	0.82 (0.66-1.01)	0.62 (0.50-0.77)
**Complete case analysis**		
Deaths/observations	709/4313	576/9004
Total protein	0.92 (0.85-0.99)	0.85 (0.78-0.92)
Animal protein	0.88 (0.80-0.96)	0.87 (0.79-0.95)
Plant protein	0.76 (0.60-0.96)	0.62 (0.49-0.78)
**Fixed-effects meta-analysis**		
Deaths/observations	838/4789	630/9610
Total protein	0.91 (0.84-0.98)	0.88 (0.82-0.95)
Animal protein	0.88 (0.81-0.95)	0.90 (0.83-0.98)
Plant protein	0.76 (0.61-0.96)	0.63 (0.50-0.80)

Cox proportional hazards regression models. Protein intake was modelled as a continuous variable (per 0.2 g/kg/day, 2% of energy intake, or 0.2 g/kg of ideal body weight/day). Hazard ratios (95% confidence intervals) were obtained from models with interaction terms between protein intake and chronic kidney disease. Models on protein sources integrated multiplicative interactions between animal protein and CKD and between plant protein intake and CKD.

© 2024 Carballo-Casla A et al. *JAMA Network Open*.

Models were adjusted for cohort (SNAC-K, Seniors-ENRICA 1, or Seniors-ENRICA 2), sex (male or female), age (years), living arrangement (alone or with spouse/partner/other), previous occupation (manual worker or non-manual worker), educational level (primary or less, secondary, or university), tobacco smoking (never, former, or current), light physical activity (daily, weekly, monthly, less often, or never), moderate-to-vigorous physical activity (daily, weekly, monthly, less often, or never), body mass index (kg/m^2), diabetes, cardiovascular disease (ischemic heart disease, cerebrovascular disease, or heart failure), chronic lung disease (chronic obstructive pulmonary disease/emphysema/chronic bronchitis or asthma), musculoskeletal disease (osteoarthritis and other degenerative joint diseases, inflammatory arthropathies, or current pathological fracture), cancer (hematological and solid neoplasms), depression and mood disorders (e.g., manic episode, bipolar affective disorder), energy intake (kcal/kg/day, kcal/day, or kcal/ideal kg/day), monounsaturated fat (g/kg/day, % of energy intake, or g/ideal kg/day), sugar (g/kg/day, % of energy intake, or g/ideal kg/day), alcohol (g/kg/day, % of energy intake, or g/ideal kg/day), and sodium intake (mg/kg/day, mg/day, or mg/ideal kg/day).

© 2024 Carballo-Casla A et al. *JAMA Network Open*.

eFigure 1. Participants' Flowchart, Stratified by Cohort

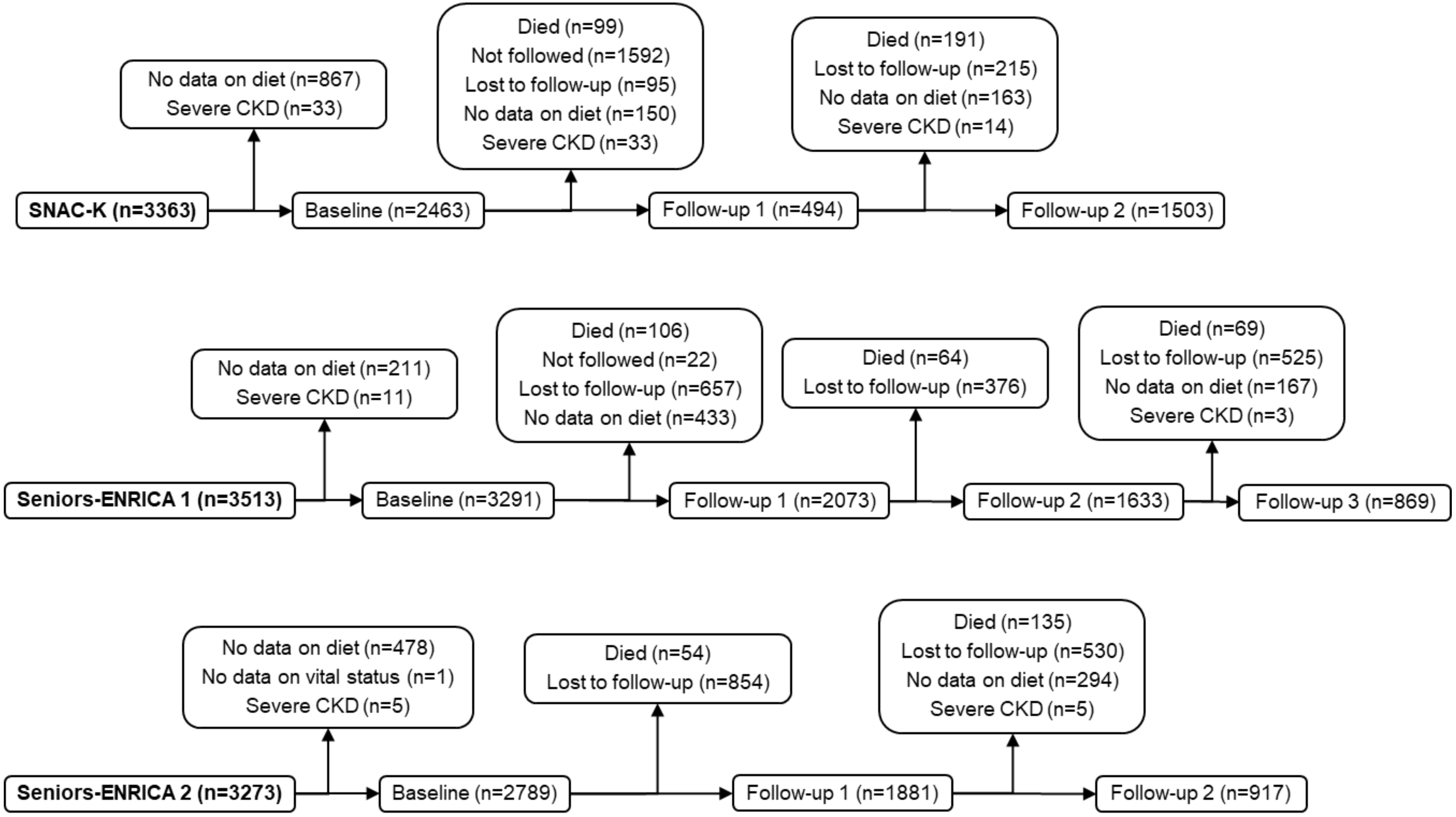

© 2024 Carballo-Casla A et al. *JAMA Network Open*.

Note that data on chronic kidney disease from Seniors-ENRICA 1's follow-up 2 were only used if study participants underwent dietary assessment at follow-up 3, while data on chronic kidney disease from Seniors-ENRICA 2's follow-up 1 were only used if study participants underwent dietary assessment at follow-up 2.

© 2024 Carballo-Casla A et al. *JAMA Network Open*.

eFigure 2. Association of Total Protein Intake With 10-Year All-Cause Mortality, Stratified by Chronic Kidney Disease and Cohort

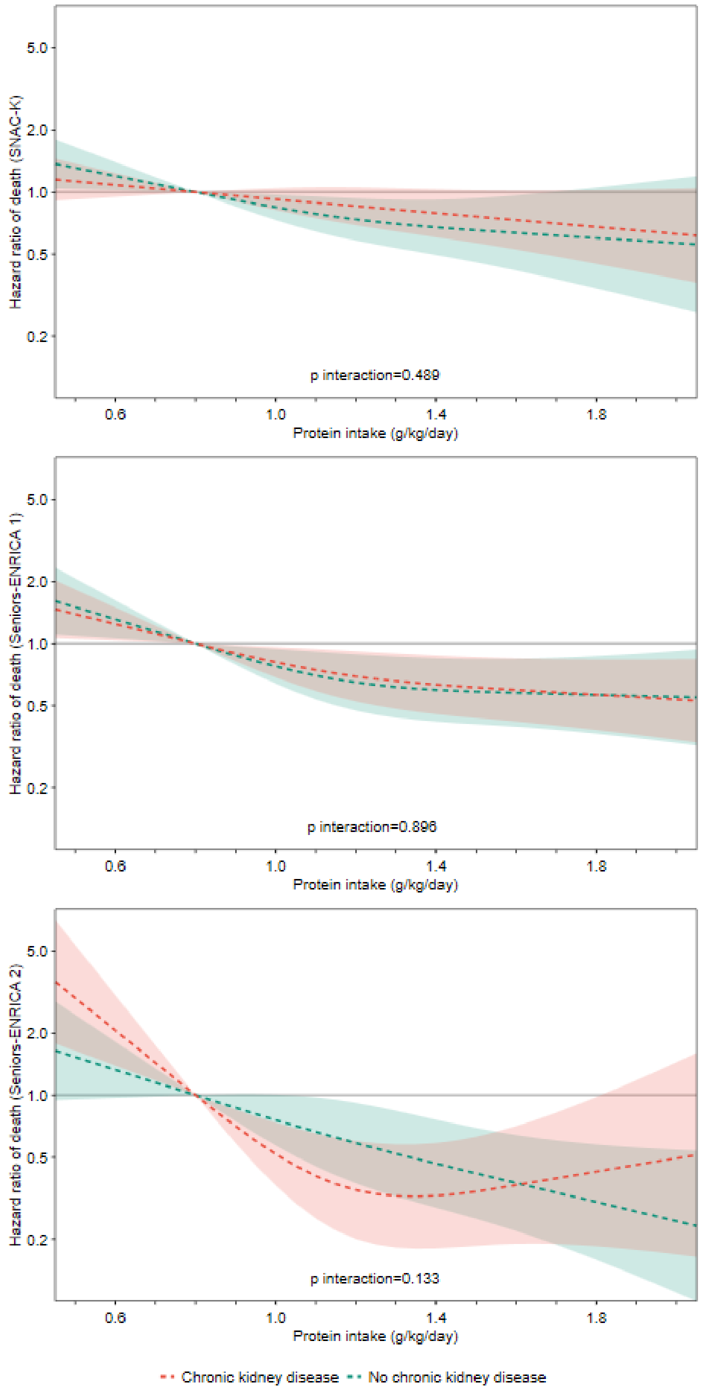

Cox proportional hazards regression models. Protein intake was modelled as a 3-knot restricted cubic spline. Hazard ratios (95% confidence intervals) were plotted for protein intakes above the 1st percentile and below the 99th percentile and obtained from models with interaction terms between

© 2024 Carballo-Casla A et al. *JAMA Network Open*.

protein intake, chronic kidney disease, and cohort (SNAC-K, Seniors-ENRICA 1, or Seniors-ENRICA 2).

Models were adjusted for sex (male or female), age (years), living arrangement (alone or with spouse/partner/other), previous occupation (manual worker or non-manual worker), educational level (primary or less, secondary, or university), tobacco smoking (never, former, or current), light physical activity (daily, weekly, monthly, less often, or never), moderate-to-vigorous physical activity (daily, weekly, monthly, less often, or never), body mass index (kg/m^2), diabetes, cardiovascular disease (ischemic heart disease, cerebrovascular disease, or heart failure), chronic lung disease (chronic obstructive pulmonary disease/emphysema/chronic bronchitis or asthma), musculoskeletal disease (osteoarthritis and other degenerative joint diseases, inflammatory arthropathies, or current pathological fracture), cancer (hematological and solid neoplasms), depression and mood disorders (e.g., manic episode, bipolar affective disorder), energy intake (g/kg/day), monounsaturated fat (g/kg/day), sugar (g/kg/day), alcohol (g/kg/day), and sodium intake (mg/kg/day).

© 2024 Carballo-Casla A et al. *JAMA Network Open*.

eFigure 3. Association of Total Protein Intake With 10-Year All-Cause Mortality, Stratified by Chronic Kidney Disease and Sex

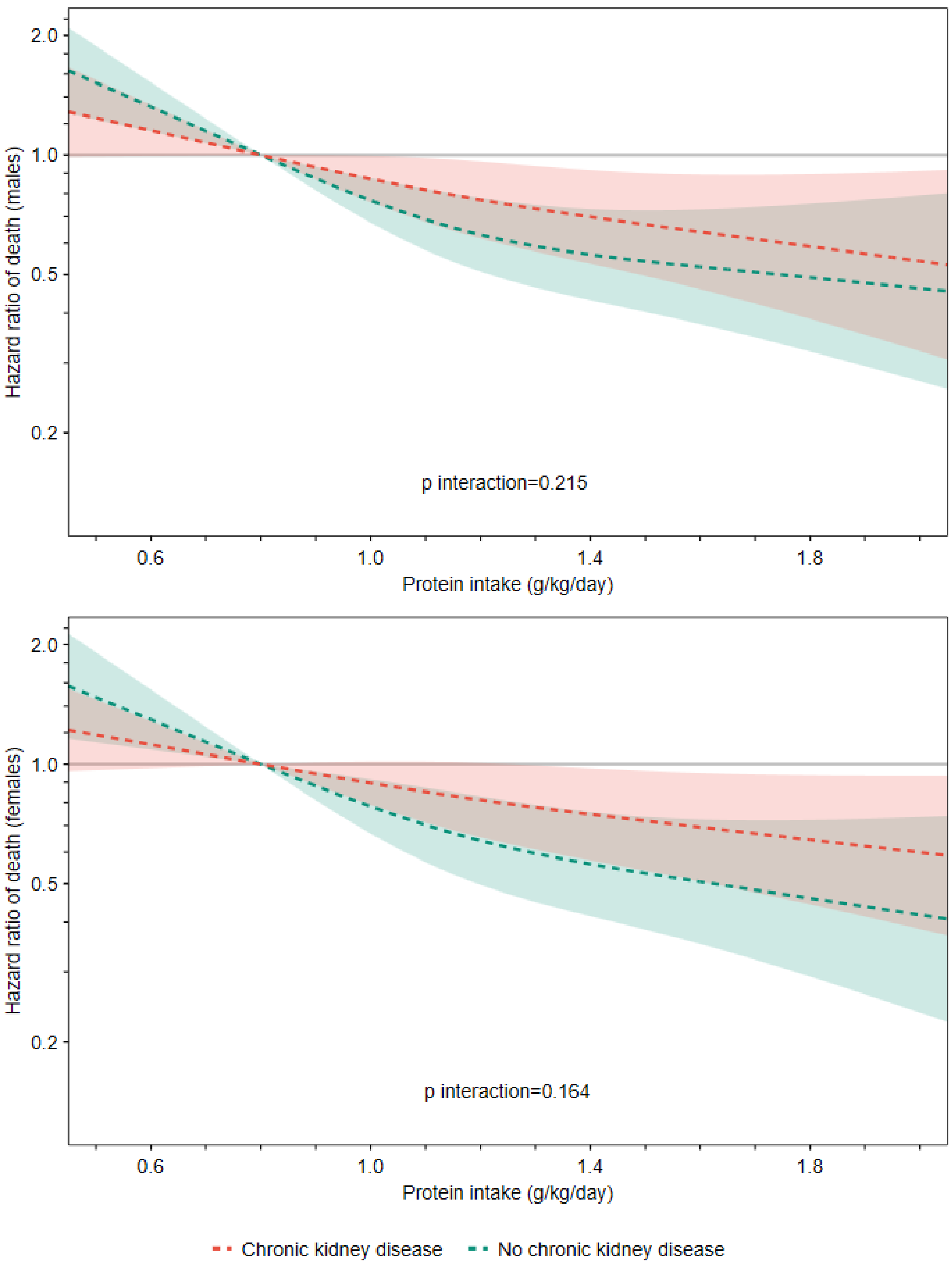

Cox proportional hazards regression models. Protein intake was modelled as a 3-knot restricted cubic spline. Hazard ratios (95% confidence intervals) were plotted for protein intakes above the 1st percentile and below the 99th percentile and obtained from models with interaction terms between protein intake, chronic kidney disease, and sex (male, female).

© 2024 Carballo-Casla A et al. *JAMA Network Open*.

Models were adjusted for cohort (SNAC-K, Seniors-ENRICA 1, or Seniors-ENRICA 2), age (years), living arrangement (alone or with spouse/partner/other), previous occupation (manual worker or non-manual worker), educational level (primary or less, secondary, or university), tobacco smoking (never, former, or current), light physical activity (daily, weekly, monthly, less often, or never), moderate-to-vigorous physical activity (daily, weekly, monthly, less often, or never), body mass index (kg/m^2), diabetes, cardiovascular disease (ischemic heart disease, cerebrovascular disease, or heart failure), chronic lung disease (chronic obstructive pulmonary disease/emphysema/chronic bronchitis or asthma), musculoskeletal disease (osteoarthritis and other degenerative joint diseases, inflammatory arthropathies, or current pathological fracture), cancer (hematological and solid neoplasms), depression and mood disorders (e.g., manic episode, bipolar affective disorder), energy intake (g/kg/day), monounsaturated fat (g/kg/day), sugar (g/kg/day), alcohol (g/kg/day), and sodium intake (mg/kg/day).

© 2024 Carballo-Casla A et al. *JAMA Network Open*.

eFigure 4. Association of the Proportion of Plant Protein With 10-Year All-Cause Mortality, Stratified by Chronic Kidney Disease and Age

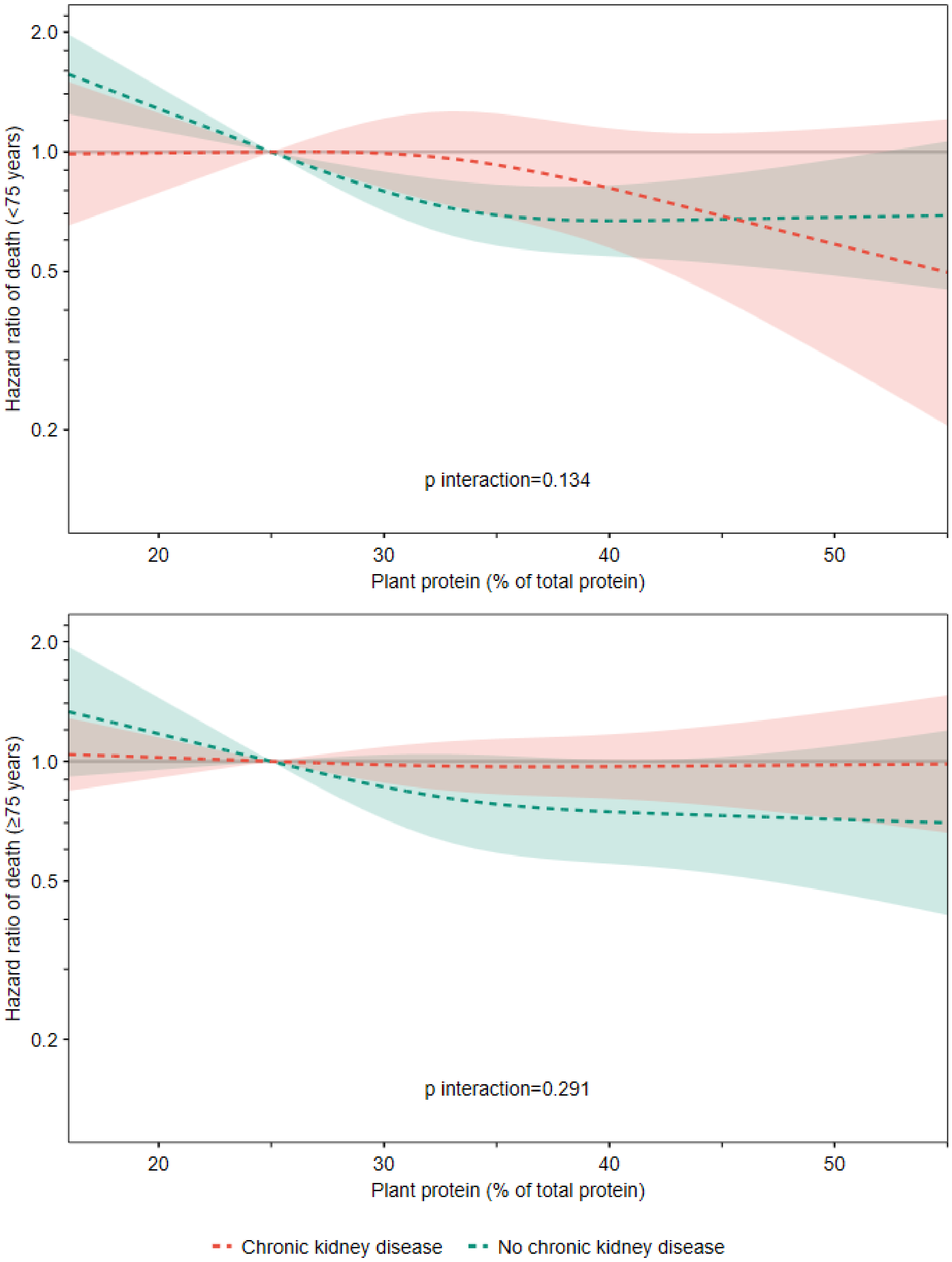

Cox proportional hazards regression models. The proportion of plant protein intake was modelled as a 3-knot restricted cubic spline. Hazard ratios (95% confidence intervals) were plotted for proportions of plant protein intake above the 1st percentile and below the 99th percentile and

© 2024 Carballo-Casla A et al. *JAMA Network Open*.

obtained from models with interaction terms between the proportion of plant protein intake, chronic kidney disease, and age.

Models were adjusted for cohort (SNAC-K, Seniors-ENRICA 1, or Seniors-ENRICA 2), sex (male or female), age (years), living arrangement (alone or with spouse/partner/other), previous occupation (manual worker or non-manual worker), educational level (primary or less, secondary, or university), tobacco smoking (never, former, or current), light physical activity (daily, weekly, monthly, less often, or never), moderate-to-vigorous physical activity (daily, weekly, monthly, less often, or never), body mass index (kg/m^2), diabetes, cardiovascular disease (ischemic heart disease, cerebrovascular disease, or heart failure), chronic lung disease (chronic obstructive pulmonary disease/emphysema/chronic bronchitis or asthma), musculoskeletal disease (osteoarthritis and other degenerative joint diseases, inflammatory arthropathies, or current pathological fracture), cancer (hematological and solid neoplasms), depression and mood disorders (e.g., manic episode, bipolar affective disorder), energy intake (g/kg/day), monounsaturated fat (g/kg/day), sugar (g/kg/day), alcohol (g/kg/day), sodium (mg/kg/day), and total protein intake (g/kg/day).

© 2024 Carballo-Casla A et al. *JAMA Network Open*.

eReferences.

1. Calderón-Larrañaga A, Vetrano DL, Onder G, et al. Assessing and Measuring Chronic Multimorbidity in the Older Population: A Proposal for Its Operationalization. J Gerontol A Biol Sci Med Sci. 2017;72(10):1417-1423. doi:10.1093/GERONA/GLW233

2. Rodríguez-Artalejo F, Graciani A, Guallar-Castillón P, et al. Rationale and Methods of the Study on Nutrition and Cardiovascular Risk in Spain (ENRICA). Rev Esp Cardiol. 2011;64(10):876-882. doi:10.1016/j.rec.2011.05.023

3. Ortolá R, García-Esquinas E, Sotos-Prieto M, et al. Mediterranean Diet and Changes in Frequency, Severity, and Localization of Pain in Older Adults: The Seniors-ENRICA Cohorts. The Journals of Gerontology: Series A. 2022;77(1):122-130. doi:10.1093/GERONA/GLAB109

4. Lagergren M, Fratiglioni L, Hallberg IR, et al. A longitudinal study integrating population, care and social services data. The Swedish National study on Aging and Care (SNAC). Aging Clin Exp Res. 2004;16(2):158-168. doi:10.1007/BF03324546/METRICS

5. Beridze G, Vetrano DL, Marengoni A, Dai L, Carrero JJ, Calderón-Larrañaga A. Concordance and Discrepancies Among 5 Creatinine-Based Equations for Assessing Estimated Glomerular Filtration Rate in Older Adults. JAMA Netw Open. 2023;6(3):e234211. doi:10.1001/jamanetworkopen.2023.4211

6. Michels KB, Willett WC. Self-administered semiquantitative food frequency questionnaires: patterns, predictors, and interpretation of omitted items. Epidemiology. 2009;20(2):295. doi:10.1097/EDE.0B013E3181931515

7. White IR, Royston P, Wood AM. Multiple imputation using chained equations: Issues and guidance for practice. Stat Med. 2011;30(4):377-399. doi:10.1002/sim.4067

© 2024 Carballo-Casla A et al. *JAMA Network Open*.

8. Fried LP, Tangen CM, Walston J, et al. Frailty in older adults: evidence for a phenotype. J Gerontol A Biol Sci Med Sci. 2001;56(3). doi:10.1093/GERONA/56.3.M146

9. Blackwell M, Iacus S, King G, Porro G. Cem: Coarsened Exact Matching in Stata. https://doi.org/101177/1536867X0900900402. 2009;9(4):524-546. doi:10.1177/1536867X0900900402

© 2024 Carballo-Casla A et al. *JAMA Network Open*.
