# Supplementary material for: Protein Intake and Mortality in Older Adults With Chronic Kidney Disease
Source: JAMA Netw Open. 2024 Aug 7;7(8):e2426577. doi: 10.1001/jamanetworkopen.2024.26577 (PMC11307132; doi:10.1001/jamanetworkopen.2024.26577)
Supplement: Supplement 2. — Data Sharing Statement [file jamanetwopen-e2426577-s002.pdf]

Data Sharing Statement

Carballo-Casla. Protein Intake and Mortality in Older Adults With Chronic Kidney Disease. *JAMA Netw Open*. Published August 07, 2024. doi:10.1001/jamanetworkopen.2024.26577

Data
Data available: Yes

Data types: Other (please specify)

Additional Information: Access to the Seniors-ENRICA and SNAC-K original data is available to the research community upon approval by the corresponding data management and maintenance committees.

How to access data: Applications for accessing Seniors-ENRICA data can be submitted to principal investigator Fernando Rodríguez-Artalejo (fernando.artalejo@uam.es). Applications for accessing SNAC-K data can be submitted to data manager Maria Wahlberg (maria.wahlberg@ki.se).

Additional Information
Who can access the data: Anyone requesting the data.
Types of analyses: Case-by-case.
Mechanisms of data availability: Case-by-case.
